# Colorectal cancer cell lines show striking diversity of their *O-*glycome reflecting the cellular differentiation phenotype

**DOI:** 10.1007/s00018-020-03504-z

**Published:** 2020-03-31

**Authors:** Katarina Madunić, Tao Zhang, Oleg A. Mayboroda, Stephanie Holst, Kathrin Stavenhagen, Chunsheng Jin, Niclas G. Karlsson, Guinevere S. M. Lageveen-Kammeijer, Manfred Wuhrer

**Affiliations:** 1 Center for Proteomics and Metabolomics, Leiden University Medical Center, Postbus 9600, 2300 RC Leiden, The Netherlands; 2 Department of Medical Biochemistry and Cell Biology, Institute of Biomedicine, Sahlgrenska Academy, University of Gothenburg, Gothenburg, Sweden

**Keywords:** *O*-Glycosylation, Glycomics, Cell lines, Colorectal cancer, Mass spectrometry, Porous graphitized carbon liquid chromatography

## Abstract

Alterations in protein glycosylation in colorectal cancer (CRC) have been extensively studied using cell lines as models. However, little is known about their *O*-glycome and the differences in glycan biosynthesis in different cell types. To provide a better understanding of the variation in *O*-glycosylation phenotypes and their association with other molecular features, an in-depth *O-*glycosylation analysis of 26 different CRC cell lines was performed. The released *O-*glycans were analysed on porous graphitized carbon nano-liquid chromatography system coupled to a mass spectrometer via electrospray ionization (PGC-nano-LC–ESI-MS/MS) allowing isomeric separation as well as in-depth structural characterization. Associations between the observed glycan phenotypes with previously reported cell line transcriptome signatures were examined by canonical correlation analysis. Striking differences are observed between the *O-*glycomes of 26 CRC cell lines. Unsupervized principal component analysis reveals a separation between well-differentiated colon-like and undifferentiated cell lines. Colon-like cell lines are characterized by a prevalence of I-branched and sialyl Lewis x/a epitope carrying glycans, while most undifferentiated cell lines show absence of Lewis epitope expression resulting in dominance of truncated α2,6-core sialylated glycans. Moreover, the expression of glycan signatures associates with the expression of glycosyltransferases that are involved in their biosynthesis, providing a deeper insight into the regulation of glycan biosynthesis in different cell types. This untargeted in-depth screening of cell line *O*-glycomes paves the way for future studies exploring the role of glycosylation in CRC development and drug response leading to discovery of novel targets for the development of anti-cancer antibodies.

**Electronic supplementary material:**

The online version of this article (10.1007/s00018-020-03504-z) contains supplementary material, which is available to authorized users.

## Introduction

With over 18 million new cases worldwide in 2018, colorectal cancer (CRC) is the third most common cancer in the world [1]. The disease is very heterogeneous with a high variability in patient prognosis and treatment response [2]. There have been various attempts to classify CRC patients into clinically relevant groups [2, 3] using single genomic markers such as microsatellite instability (MSI), BRAF and KRAS mutations. While these markers can give insights into disease processes, they cannot provide a full understanding of the molecular pathology and prediction of patient outcome [4]. Therefore, the CRC Subtyping Consortium (CRCSC) has recently made a systematic comparison of gene mutation and gene expression of primary tumours from a large set of samples and defined four subtypes of CRC with different clinical and molecular markers [4]. Consensus molecular subtype (CMS)1 tumours show prevalence of MSI together with high immune infiltration in the tumour microenvironment, associated with a better patient prognosis [3]. In contrast, the mesenchymal CMS4 tumours are characterized by infiltration with cancer-associated fibroblasts and upregulation of epithelial to mesenchymal transition (EMT) resulting in worse overall patient prognosis [3]. Both CMS2 and CMS3 tumours show strong epithelial differentiation signatures, with characteristic metabolic pathway dysregulation in the CMS3 group [4]. Although the proposed classification provides a deeper understanding of CRC and its differential molecular signatures, it is not yet clear which features will be relevant for accurate patient stratification. To design subtype-specific therapeutic strategies, translation of the CMS classification to preclinical models is needed, to enable large-scale drug screenings.

Various genetic studies have confirmed that cancer cell lines recapitulate the molecular features of the tumours [5, 6] and the same has been confirmed for CMS [7]. Recently, profiling of 34 CRC cell lines revealed consistency at the gene, microRNA and protein levels, dominated by two distinct clusters. The colon-like cluster has high expression of gastrointestinal specific markers, while the second cluster contains undifferentiated cell lines showing upregulation of transforming growth factor (TGF)-β-induced genes and EMT signatures [8]. These two groups significantly associate with CMS groups, where CMS1 and CMS4 cluster as undifferentiated, and CMS2 and CMS3 as colon-like [8].

Next to genetic, metabolic and proteomic signatures, protein glycosylation is a major factor in colon differentiation and CRC development [9]. It has been shown that malignant transformation changes the glycosylation machinery of the cells, affecting the function of the oncogenic receptors that are involved in the control of cell proliferation and differentiation [10]. Moreover, glycan binding proteins, expressed by immune cells in the tumour microenvironment, respond to these changes, often resulting in an immunosuppressive response [11]. Therefore, unravelling glycan-based interactions in cancer is instrumental for disclosure of molecular mechanisms underlying cancer biology.

Due to their continuous availability, cell lines are often used as models for studying glycosylation changes in cancer. Recently, the *N*-glycosylation of a set of CRC cell lines has been characterized, revealing association of antennary fucosylation with differentiation and caudal type homeobox 1 (CDX1) expression [12, 13]. Another major class of colon glycans is mucin-type *O*-glycans, mainly carried by heavily glycosylated mucin proteins, which are the major components of the mucus layer in the gastrointestinal tract. Mucin type O-linked glycosylation is initiated by the transfer of *N*-acetylgalactosamine (GalNAc) to Ser/Thr of both mucin and non-mucin glycoproteins which are shown to be altered in various cancers including CRC [14, 15]. Unfortunately, little is known about *O-*glycosylation of cell lines due to its complexity, the presence of multiple isomeric structures as well as the lack of enzymatic release methods, making it overall a challenging task [14].

Here, we present an in-depth structural analysis of *O*-glycosylation phenotypes of 26 CRC cell lines derived from both primary tumours and metastatic sites. We optimized a 96-well plate PVDF membrane-based method [12] for preparation of released *O-*glycans from 500,000 cells via reductive beta-elimination [16]. Released *O*-glycans were analysed on a sensitive analytical platform, namely, porous graphitized carbon nano-liquid chromatography coupled to a tandem mass spectrometer (PGC nano-LC–ESI-MS/MS) using negative electrospray ionization. Major differences are observed between 26 analysed CRC cell lines, revealing the diversity of the CRC cell line *O-*glycome. Moreover, associations are found between the observed glycome phenotypes and cell line gene expression as well as their differentiation.

## Materials and methods

### Cells and cell culture

Human CRC cell lines were obtained from the Department of Surgery of the Leiden University Medical Center (LUMC), Leiden, The Netherlands, as well as the Department of Pathology of the VU University Medical Center (VUmc), Amsterdam, The Netherlands. Further details are provided in Supplementary file 1.

### *O*-Glycan release and analysis

Lysed cell pellets containing 500,000 cells were loaded to the preconditioned PVDF membrane plate wells and denatured with guanidine hydrochloride and dithiothreitol (DTT) at 60 °C. After removing the denaturation agent, *N*-glycans were released by PNGase F digestion overnight at 37 °C. Upon removal of *N*-glycans, 50 μL of 0.5 M sodium borohydride (NaBH_4_) in 50 mM potassium hydroxide (KOH) was added to each well and incubated for 16 h at 50 °C for the release of *O*-glycans via reductive beta-elimination. Desalting of the samples was performed using a cation exchange resin Dowex 50 W X8 which was self-packed into 96-well filter plates. Desalted *O*-glycans were further purified via solid phase extraction by packing bulk sorbent carbograph slurry into 96-well filter plates. Analysis was performed using a PGC nano-LC–ESI-MS/MS platform. More details are provided in Supplementary file 1.

### Glycan structure analysis and relative quantification

Identification of glycans was performed based on PGC retention time, known biosynthetic pathways, and manual inspection of fragmentation spectra following known MS/MS fragmentation pathways of *O*-glycan alditols in negative-ion mode [17, 18]. Glycan sequences and linkages were confirmed by the analysis of glycans upon α2-3 neuraminidase, α1-3/4 fucosidase, and β1-4 galactosidase digestion. Relative quantitation was performed on the total area of all *O*-glycans within one sample normalizing it to 100%. MS/MS mass lists were exported from the DataAnalysis software for upload to Unicarb DR repository [19]. A more detailed description is provided in Supplementary file 1.

### Statistical analysis

An imputation of the minimum positive number (0.0001) was performed to enable use of the statistical tools sensitive to the missing values such as principal component analysis (PCA). Regularized canonical correlation analysis was performed using rcc function as it is implemented in the “mixOmics” package [20]. Data analysis and visualization were performed in ‘’R’’ software.

## Results

### High-throughput and robust *O*-glycan release from cells

To analyse the *O*-glycosylation of 26 CRC cell lines, we established a high-throughput sample preparation in 96-well format (Supplementary Figure S1). The combination and optimization of two previously established protocols [12, 16] allowed sequential release of both *N-* and *O-*glycans from cell lysates in a higher-throughput manner using 96-well plates. *O*-glycans were analysed on PGC nano-LC–ESI-MS/MS platform which revealed 178 different *O*-glycan structures. Of these, 153 passed the quality control criteria and were included in the analysis (Supplementary Figure S3 and Supplementary Table 1: S3–S31). To assess the technical and biological variation of *O*-glycan profiles for each cell line, the complexity of each sample was reduced by compiling the relative peak areas for all glycans to single mass spectrometry average compositions (MSAC) [21] (Supplementary Table 1: S1) representing the normalized number of sugar residues and modifications per glycan molecule. Low technical variability of our workflow is illustrated by the close clustering of scores in the PCA model from two technical replicates of each cell line as well as the triplicate of *O-*glycans released from bovine fetuin (standard) (Supplementary Figure S2). In addition, the close clustering scores from the cell lines which were cultured and analysed in three biological replicates (HT29, HCT116, SW480, SW620, and HCT8) revealed a low biological variability (marked as A, B and C in the respective PCA plot). The highest variability in glycosylation profiles was observed in cell lines SW480, HT29 and HCT116, which may be attributed to the fact that the replicates for these cell lines were prepared at different sites (VUmc and LUMC) as well as using different media (detailed description in Supplementary file 1).

### High diversity of CRC cell lines *O-*glycosylation profiles

**Fig. 1:**
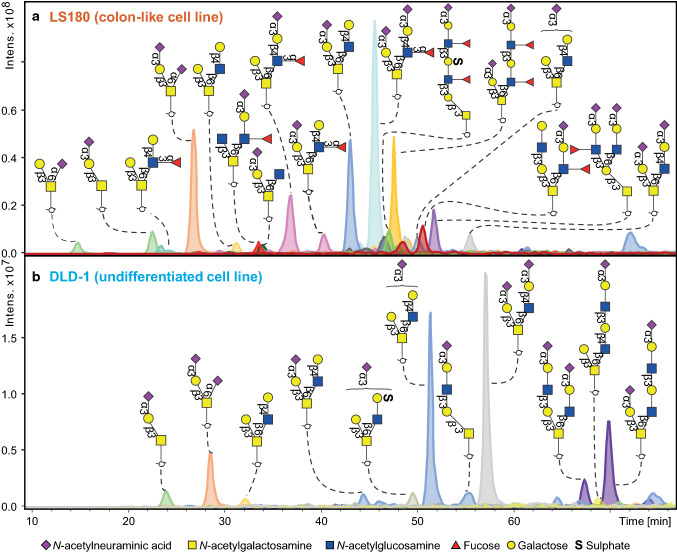
*O*-glycan profiles from two exemplary CRC cell lines. **a** *O-*glycan profile of the mucin-secreting well-differentiated (colon-like) cell line LS180 is characterized by high expression of sialyl Lewis x/a and Lewis x/a antigens. These antigens were found to be present on core 1, core 2 and core 4 glycan structures, both in linear and I-branched form. In contrast, **b** displays poorly differentiated human colon adenocarcinoma cell line DLD-1, showing an *O-*glycan profile which is dominated by core 2 sialylated glycans lacking any fucosylated antigens

The comparison of the 26 CRC cell lines revealed striking differences between the *O*-glycomes (Supplementary Figure S3). Many, yet undescribed, *O*-GalNAc-linked glycans were detected varying in size from 2 up to 14 monosaccharide residues. An illustration of the diversity between cell lines is shown in Fig. 1. The top panel shows the glycan profile of the mucin-secreting cell line LS180 (human colon adenocarcinoma) which is characterized by high expression of sialyl Lewis x/a and Lewis x/a antigens. These antigens were found to be present on core 1, core 2 and core 4 glycan structures, both in linear and I-branched form. In contrast, DLD-1 (bottom panel), a poorly differentiated human colon adenocarcinoma cell line, showed an *O*-glycan profile which is dominated by core 2 sialylated glycans lacking any fucosylated antigens.

Only three glycans were present in all samples, albeit at highly varying abundances: disialyl-T antigen (NeuAcα2-3Galβ1-3(NeuAcα2-6)GalNAcol), the α2,3 sialyl-T antigen (NeuAcα2-3Galβ1-3GalNAcol), and the di-sialylated core 2 glycan (NeuAcα2-3Galβ1-3(NeuAcα2-3Galβ1-4GlcNAcβ1-6)GalNAcol). The main core structures in most cell lines were core 2, followed by elongated structures of core 1. Core 3 structures were only detected in low amounts, while core 4 structures were expressed the most in HCT-15, LOVO and SW1116 cell lines (Supplementary Figure S4). Interestingly, all cell lines were dominated by sialylated glycan species (Supplementary Figure S4). *N*-acetylneuraminic acids (NeuAc) on *O*-glycans were mostly α2,3-linked to a galactose residue. Moreover, α2,6-linked sialylation was observed on the innermost GalNAc predominantly in the context of sialyl-T and disialyl-T antigens. Cell lines HT29 and WiDr showed the highest expression of α2,3-sialylated *O*-glycans (Supplementary Table 2: S2). In addition, 42 *O*-glycans were found to contain sulphate modifications mainly expressed by SW948 and LS411N cell lines (Supplementary Table 2: S2).

**Fig. 2:**
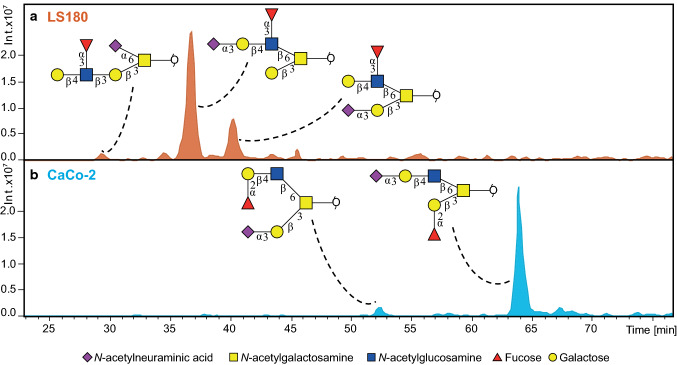
Chromatographic separation of *O*-glycan isomers at *m/z* 1186.40 [M–H]^−^ carrying different antigens. The extracted ion chromatograms show a different retention behaviour of Lewis antigen isomers in the **a** LS180 cell line. Namely, core 1 carrying Lewis x antigen with α2,6-linked NeuAc linked to the core GalNAc (RT = 30.2 min), core 2 glycan carrying sialyl-Lewis x antigen on the 6 arm (RT = 37.2 min), and core 2 glycan carrying Lewis x antigen on the 6 arm (RT = 40.7 min). Additionally, the **b** CaCo-2 cell line illustrates the separation of core 2 blood group antigen H type 2 (RT = 52.4 min), and a core 2 mucin blood group antigen H type 3 (RT = 63.8 min)

A major advantage of PGC chromatography is the high separation power that enables discriminating between glycan linkages and positional isomers [17, 18]. Identification of *O*-glycans was performed based on PGC retention time, described biosynthetic pathways and manual inspection of fragmentation spectra following known MS/MS fragmentation patterns of *O*-glycan alditols in negative-ion mode [17, 22]. All annotated structures are listed in Supplementary Table 2: S1. MS/MS peak lists with glycan annotations per cell line are available via an online repository Unicarb DR [19] (https://unicarb-dr.biomedicine.gu.se/). Figure 2 shows the powerful chromatographic separation of five glycan isomers with the same composition H2N2F1S1 in both LS180 and CaCo-2 cell lines. Glycan sequences and linkages were confirmed by the analysis of glycans upon α2-3 neuraminidase digestion, as well as additional combined α1-3/4 fucosidase and β1-4 galactosidase digestion as demonstrated by Supplementary Figures S5, S6 and S7. With this approach, we were able to identify the most abundant glycans representing more than 95% of the relative intensity for 13 cell lines, and over 90% of the relative intensity for the remaining 13 cell lines.

### Glycan traits are associated with cell line differentiation

**Fig. 3:**
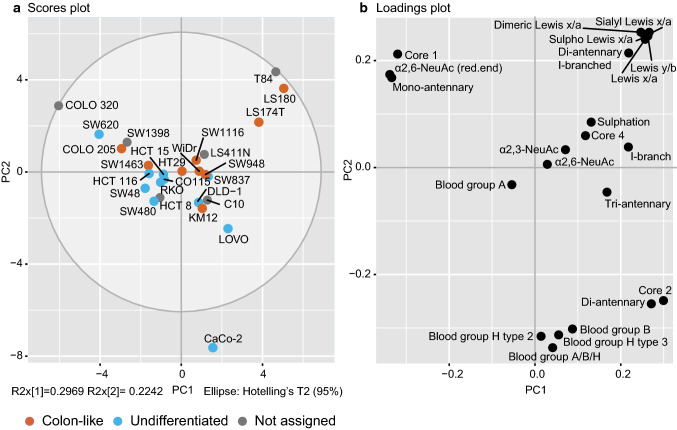
PCA based on relative abundance (%) of calculated structural glycan features. A separation between colon-like and undifferentiated cell lines is illustrated in the **a** PCA score plot of PC1 against PC2. **b** The PCA loading plot displays the variables that drive the separation in the PCA model. The top three principal components explain 68.58% of the variation within the data. Biological and technical replicates were averaged per cell line

**Fig. 4:**
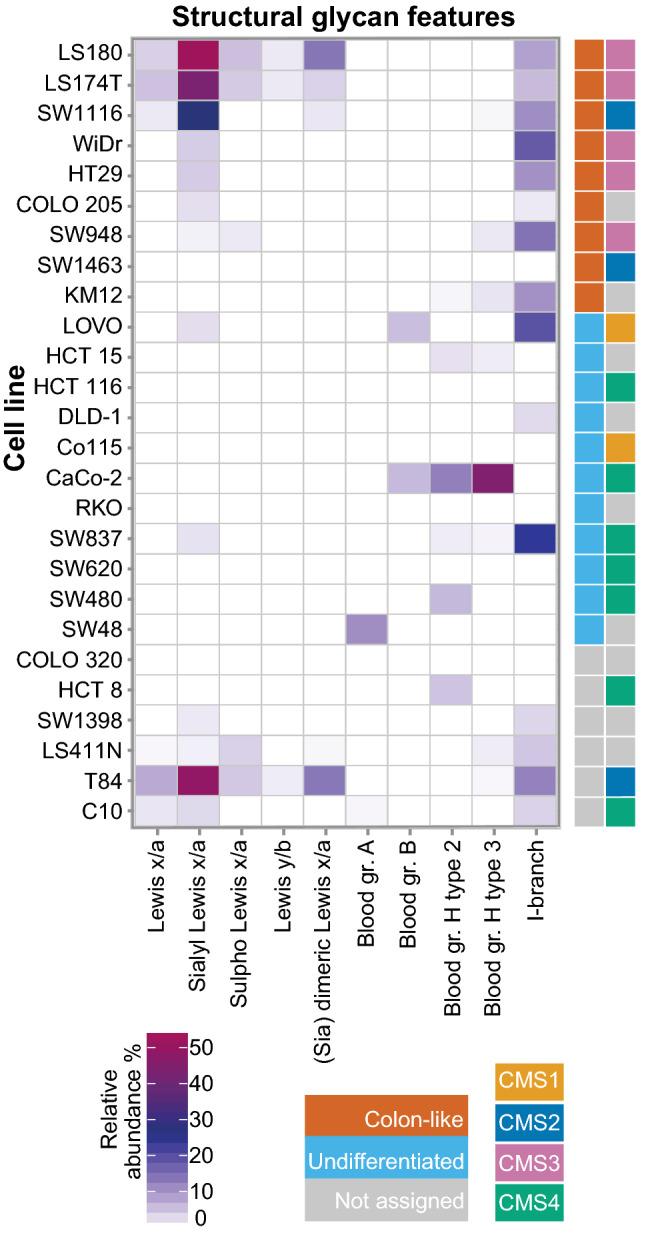
Relative abundance of structural glycan features per cell type. Geometrical tile of the relative abundance (%) of the calculated structural glycan features (*x* axis) and cell line type (*y* axis). Different classifications of the cell lines are displayed with colour codes based on gene expression (colon-like in red and undifferentiated in light blue) and consensus molecular subtypes (CMS1 in yellow, CMS2 in dark blue, CMS3 in pink, and CMS4 in green). Non-assigned cell lines were marked in grey for the gene expression as well as for the CMS status

To explore the specific *O-*glycan phenotypes in CRC more in-depth, the assigned glycans were relatively quantified and grouped based on glycan structural features such as core (1, 2, 3, or 4), I-branch (GlcNAcβ1-6Gal-R), α2,3- or α2,6-sialylation, Lewis x/a (Galβ1-4/3(Fucα1-3/4)GlcNAc-R, sialyl Lewis x/a (NeuAcα2-3Galβ1-4/3(Fucα1-3/4)GlcNAc-R, (sialyl) dimeric Lewis x/a (NeuAcα2-3Galβ1-4/3(Fucα1-3/4)GlcNAcβ1-3Galβ1-4/3(Fucα1-3/4)GalNAc-R, blood group A GalNAcα1-3(Fucα1-2)Galβ1-3/4-R, blood group B (Galα1-3(Fucα1-2)Galβ1-3/4-R, blood group H type 3 (Fucα1-2Galβ1-3(R-)GalNAcol) and blood group H type 2 (Fucα1-2Galβ1-4GlcNAc-R) (Supplementary Table 2: S2)*.* The structures that could not be unambiguously assigned were not included in the calculations of the structural features. The obtained CRC *O*-glycosylation signatures were further explored by PCA (Fig. 3). Analysis of the score and loadings plot (Fig. 3a, b) of the model shows that HT29 and its derivative WiDr cell line are positioned close to the centre of the Hotelling circle and as such could be viewed as the examples of the average glycosylation profile. The cell lines derived from the same patient (HCT15, HCT8 and DLD-1) could also be considered as the examples of the average profile. Yet, a distance between them indicates the differences in their *O-*glycomes. This is supported by the Fig. 4 where DLD-1 cell line shows higher expression of I-branched glycans, and no expression of blood group antigen H, while the HCT8 and HCT15 express glycans carrying blood group H antigens. Moreover, another two cell lines derived from the same patient, SW480 from primary tumour and SW620 from lymph node metastasis do not cluster together on the score plot. Here, differences are found in the expression of blood group antigen H carrying glycans, which are present in higher levels in the SW480 cell line, while SW620 does not express any fucosylated epitopes. Finally, the model reveals a similarity in the glycosylation profiles of the closely positioned cell line variants LS180 and LS174T on the outskirts of the score plot, as a result of very high expression of Lewis x/a antigens.

**Fig. 5:**
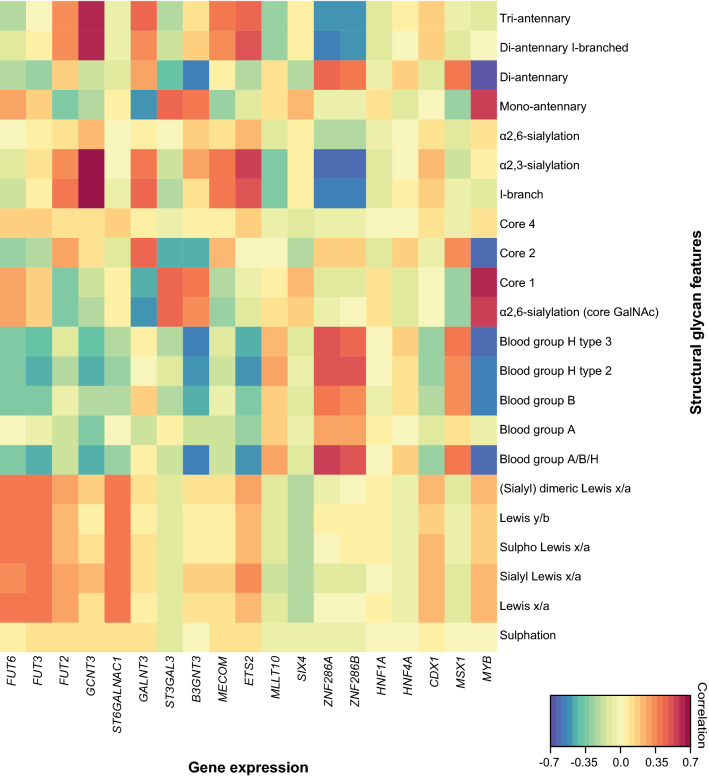
Associations of structural glycan features with gene expression. The clustered image map of the canonical model displaying associations of glycan structural features with gene expression of relevant GTs and transcription factors differentially expressed in colon-like vs undifferentiated cell lines [8]

Differentiation phenotype of the cells (colon-like and undifferentiated) [8] appears as a visible trend separating the first two components of the score plot (Fig. 3a), while no association could be found with other cancer cell characteristics (e.g., MSI, oncogene mutations, tumour stages; data not shown). To decipher which structural glycan features drove the separation in the PCA model, we explored the variables in the PCA loadings plot (Fig. 3b). Cell lines clustering in the left part of the score plot in Fig. 3a (Colo320, SW620, SW1398, SW48, SW480, HCT116, RKO, Co115, and HCT15) have higher expression of core 1 and α2,6-linked NeuAc linked to the core (Fig. 3b) which is also displayed in Supplementary Figure S4. Most of the cell lines in this cluster have been previously classified as undifferentiated based on low expression of gastrointestinal specific genes [8]. On the other hand, mucin-secreting cell lines LS180, LS174T [23] and T84 [24] are characterized by abundant and diverse glycosylation with very high expression of Lewis antigens clustering in the upper right part of the PCA score plot. In contrast, CaCo-2 cell line displays a different phenotype, rich in blood group H type 2 and type 3 antigens, as well as Sd^a^ (GalNAcβ1-4(NeuAcα2-3)Galβ-) and Cad (GalNAcβ1-4(NeuAcα2-3)Galβ1-3(NeuAcα2-6)-) antigens. Colon-like cell lines show higher expression of Lewis-like antigens, predominantly sialyl Lewis x/a epitopes (Fig. 4) which could be a trait of the CMS3 metabolic subtype (represented by LS180, LS174T, HT29, WiDr and SW948). The I-antigen branching is found more often in the colon-like cell lines (Fig. 5) such as WiDr, SW948, SW1116 and HT29. However, one of the undifferentiated cell lines, SW837, shows the highest expression of I-branched glycans. Most of the CMS4 cell lines are characterized by a high level of overall α2,3-sialylation, no expression of Lewis antigens (with the exception of SW837 and C10 cell lines), together with relatively higher expression of blood group antigen type 2.

### Glycomic signatures associate with cell line transcriptomics

To understand which genes underlie the glycosylation phenotypes observed in this study, we performed a canonical correlation analysis using mRNA expression data from an online available dataset [8]. Primarily, we selected genes involved in the biosynthesis of *O*-glycans which showed the largest fold change in expression (log_e_ > 0.5) when comparing colon-like and undifferentiated cell lines with high statistical significance (Bonferroni corrected *p* value < 0.05), displayed in the volcano plot (Supplementary Figure S8). Colon-like cells show higher expression of the following genes encoding for glycosyltransferases (GTs): *B3GNT3, FUT2, FUT3, FUT6, GALNT3, GCNT3,* and *ST6GALNAC1.* In contrast, the undifferentiated cell lines show a slightly higher expression of *ST3GAL3.* The correlations of glycan structural features with expression of the selected genes are illustrated in the clustered image map of the canonical model (Fig. 5). A moderate correlation between the *FUT3* and Lewis x/a (*r* = 0.40) as well as between *FUT6* and sialyl Lewis x/a epitopes (*r* = 0.36) is observed. *ST6GALNAC1* gene expression also correlates with the expression of sialyl Lewis x/a (*r* = 0.38) and Lewis x/a epitopes (*r* = 0.40). Additionally, *GCNT3* shows a strong correlation with α2,3-sialylation (*r* = 0.70) and I-branching (*r* = 0.66) reflecting the expression of sialylated I-branched diantennary and triantennary *O*-glycans. *GALNT3* encoding for *O-*glycosylation initiating enzyme shows positive correlation with core 2 (*r* = 0.43), I-branching (*r* = 0.44), and α2,3-sialylation (*r* = 0.38), as well as a negative correlation with α2,6-core sialylation (*r* =  − 0.50), and core 1 glycans (*r* =  − 0.45). *B3GNT3*, a gene encoding for β-1,3-*N*-acetylglucosaminyltransferase 3 and involved in the biosynthesis of poly-*N*-acetyllactosamine chains, is found to be negatively correlated with the expression of blood group A, B or H (blood group A/B/H) antigens (*r* =  − 0.57), core 2 glycans (*r* =  − 0.45) and shows a positive correlation with core 1 glycans (*r* = 0.40). *ST3GAL3* is the only gene involved in the *O-*glycan biosynthesis which shows higher expression in the undifferentiated cell lines, displaying correlation with core 1 (*r* = 0.45) and α2,6-sialylated glycan expression (*r* = 0.46).

### Transcriptional regulation of glycosyltransferase expression

To gain more insight into the regulation of *O*-glycosylation, we also examined the associations of glycan epitopes with transcription factors which showed the highest fold change in expression (log_e_ > 0.5) when comparing colon-like and undifferentiated cell lines [8] with high statistical significance (Bonferroni corrected *p* value < 0.05) (Supplementary Figure S8). The colon-like cells show significantly higher expression of the following transcription factors: *CDX1, ETS2, HNF1A, HNF4A, MECOM* and *MYB.* In contrast, the undifferentiated cell lines showed elevated expression levels of the following transcription factors: *MLLT10, MSX1, SIX4, ZNF286A* and *ZNF286B*. The clustered image map of the canonical model (Fig. 5) displays the moderate positive correlation between the expression of Lewis antigens and transcription factors *CDX1* and *ETS2*. Relatively strong correlation is seen for *ETS2* with α2,3-sialylation (*r* = 0.57) and I-branching (*r* = 0.51). The *MYB* gene, which is highly expressed in colon-like CRC cells, showed a strong positive correlation with the expression of core 1 (*r* = 0.59) and α2,6-sialylated glycans (*r* = 0.57) and a strong negative correlation with blood group A, B or H (Blood group A/B/H) carrying structures (*r* =  − 0.62). On the other hand, two transcription factors *ZNF286A* and *ZNF286B,* expressed in undifferentiated cell lines, show high negative correlation with α2,3-sialylation (*r* =  − 0.63, and *r* =  − 0.60, respectively) and I-branching (*r* =  − 0.55 and *r* =  − 0.53, respectively), together with a positive correlation with blood group antigen A, B or H carrying glycans (*r* = 0.58 and *r* = 0.53, respectively). To identify the possible transcriptional regulation of specific GTs, we tested the associations between transcription factor expression and GT expression (Supplementary Figure S9). In contrast with the rather weak correlations of *MYB* expression and Lewis antigen expression, *MYB* expression does show correlation with *FUT3* and *B3GNT3* genes. We also observe strong negative correlations between the expression of *ZNF286A* and *ZNF286B* genes with the expression of *GCNT3* and *B3GNT3* genes involved in the elongation and branching of *O*-glycan molecules next to a positive correlation with the expression of *ST3GAL3*.

## Discussion

In the present study, we investigated the *O-*glycosylation phenotypes of 26 CRC cell lines derived from both primary tumours and metastatic sites, revealing pronounced differences between the cell lines. The PGC nano-LC–MS/MS platform allowed separation of isomeric *O*-glycan species which were structurally elucidated relying on negative mode tandem mass spectra and exoglycosidase treatment. Using this approach, we were able to create a detailed CRC cell line *O*-glycan MS/MS spectral library, which will be available via https://unicarb-dr.biomedicine.gu.se/, serving as an important resource, leading towards automated *O*-glycan identification via spectral matching tools.

**Fig. 6:**
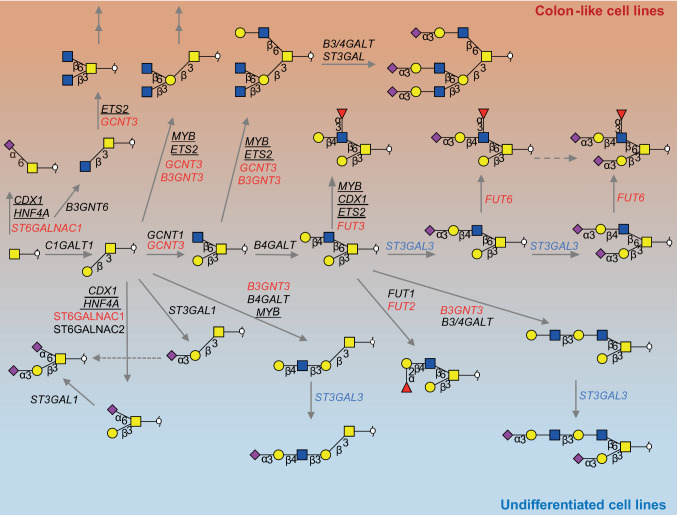
Proposed model explaining the differences in glycosylation between colon-like and undifferentiated cell lines. The most abundant structures in colon-like and undifferentiated cell lines and their biosynthesis pathways with genes encoding for the GTs involved in the biosynthesis. GTs upregulated in colon-like cell lines are marked in red, whereas genes upregulated in undifferentiated cell lines are marked in blue. Double arrows indicate structures that can be elongated further by action of different GTs. Transcription factors showing correlation with the expression of GTs are underlined. Please note that other GTs could also be involved in the biosynthesis of the displayed glycans

An exploratory, qualitative analysis of the entire pool of our data clearly shows a difference in *O*-glycome profiles between the colon-like well-differentiated cell lines and undifferentiated ones. A proposed model explaining the differences in glycosylation and biosynthesis in different cell types is displayed in Fig. 6. For instance, the well-differentiated cell lines show an overall higher expression of Lewis antigens and I-branched glycans, while the undifferentiated cell lines show a higher abundance of glycans carrying an α2,6-linked NeuAc to the core GalNAc. Few exceptions could also be observed. For instance, Colo205, a colon-like but also a metastatic cell line [25], clusters together with the undifferentiated cell lines expressing higher relative amounts of core 1 α2,6-sialylated glycan species. Similarly, the rectal adenocarcinoma SW1463 cell line [26] does not show expression of Lewis antigens characteristic for other mucus producing cell lines. Such observations can be valuable on their own, but offer only a limited space for a functional interpretation of the data. Taking advantage of the existing pool of published transcriptomics data on the selected cell lines, we attempted to generate regulational hypotheses using the correlations between the *O*-glycan profiles and the transcripts most strongly associated with the differentiation status of the studied cell lines. Associations were found between gene expression and cell line differentiation based on the *O*-glycome profiles of 26 CRC cell lines. Here, Lewis antigen expression was found to be the most abundant in the mucin-secreting cell lines LS174T [23], LS180 and T84 [24], correlating with the expression of genes *FUT3* and *FUT6* encoding for the GTs involved in the biosynthesis of the Lewis antigens. Expression of Lewis antigens was also associated with *ST6GALNAC1* gene expression encoding for an α2,6-sialyltransferase which acts on the core GalNAc residue. Although we have detected sialyl-Tn antigen in the mucin-secreting cell lines (LS180 and LS174T), we could not quantify mono- and disaccharide *O*-glycan alditols in a reliable manner due to limitations of the method. Previously, sialyl-Tn antigen was found to be highly expressed in LS174T cell line and also showed correlation with upregulation of *ST6GALNAC1* [27]. In concordance with previous studies, demonstrating an association of CDX-1 transcription factor with the expression of GTs *FUT3* and *FUT6* and expression of multifucosylated *N*-glycans in cell lines [12, 13], a correlation between Lewis antigen-expressing cell lines and *CDX1* expression was observed. Moreover, some cell lines with high expression of α2,3-sialylated and multifucosylated *N*-glycans [12] (HT29, WiDr, T84, and LOVO) also revealed a high expression of *O-*glycans carrying sialyl Lewis x/a antigens. Strikingly, the cell lines with the highest expression of sialylated Lewis x/a type antigens on *O-*glycans (LS180, LS174T and SW1116) express mainly non-sialylated Lewis antigens on *N-*glycans [12]. These findings emphasize the importance of studying glycosylation in a glycan type-specific manner, as they can exhibit different glycan motifs and may convey different functions.

In contrast to high Lewis antigen and I-branching signatures of colon-like cell lines, the undifferentiated cell lines show a high abundance of core 1 glycans carrying an α2,6-linked NeuAc to the core GalNAc. This signature mainly reflects the high relative abundance of the ubiquitous disialyl-T antigen compared to a lower abundance of elongated and branched *O*-glycans. The addition of NeuAc in the α2,6-position of the Tn or T antigen will prevent formation of different core structures and further elongation of cores with different structural epitopes [28]. This may explain the absence of core 3 structures in cell lines, which similarly to inflamed colon tissue [21], show low abundance of core 3 structures compared to normal colon tissue. Some reports have shown that upregulation of *ST3GAL3*, *ST6GALNAC1* and *ST6GALNAC2* could be associated with invasion to lymph nodes and poor patient prognosis [29]. Furthermore, enhanced sialylation of tumour cells can engage inhibitory sialic acid binding lectin-Siglec receptors on natural killer (NK) cells, providing an immune evasion mechanism [11]. The competition between different GTs for the core GalNAc residue can explain the strong inverse correlations between core 2 and α2,6-sialylation observed in our data (Fig. 6). Competition involved in core 2 glycan biosynthesis such as *GCNT1*, *GCNT3* and *ST6GALNAC1* have been described extensively in the literature [30, 31]. The *GCNT3* gene encodes a mucin type β1,6-*N*-acetylglucosamine transferase, which adds an *N*-acetylglucosamine (GlcNAc) to the core GalNAc forming core 2 or core 4 structures. Importantly, *GCNT3* also has I-antigen biosynthetic activity, by adding the residue to the terminal galactose, forming another branching point. *GCNT3* expression has been associated with reduction of proliferation and invasion as well as with higher sensitivity to the chemotherapeutic 5-fluorouracil [32]. Low *GCNT3* expression has been proposed as a prognostic marker that could be used to identify early-stage colon cancer patients at high risk of relapse [33]. It has been demonstrated that the expression of *GCNT3* is higher in the colon-like cell lines [8], showing a strong correlation with the expression of I-branched glycans as well as with α2,3 sialylation in our data. However, one of the undifferentiated cell lines SW837, derived from the rectum [34], shows the highest expression of I-branched glycans.

Our results also show expression of histo-blood group ABH antigens carried by *O*-glycans mainly from undifferentiated cell lines. Apart from the erythrocytes, the ABH antigens can be expressed also in the gastrointestinal tract and secretions, where the *Se-*FUT enzyme (*FUT2*) is responsible for their biosynthesis [35]. However, several reports have demonstrated the expression of blood group ABH antigens in colon cancers from non-secretors with inactive enzyme [36, 37]. Cell lines showing expression of blood group H antigens such as CaCo-2, HCT15, HCT8, SW948, SW1116, and SW837 were derived from tumours of blood group O secretor positive individuals [38]. We have also observed the expression of blood group B antigens by the CaCo-2 cell line, and this incompatible expression in the gut of blood group O individuals has been previously observed in colon cancer [39]. LOVO cell line, derived from a B blood type individual, expressed the blood group B carrying glycans; however, the SW48 (AB blood group individual) shows expression of only blood group A glycans. Cell lines SW480, SW620 and HT29 derived from blood group A individuals do not show expression of the A antigen carried by *O*-glycans, although these cell lines have shown a substantial A transferase activity [38]. HCT8 cell line derived from the ileocecal colon shows higher expression of blood group antigens H type 2, compared to HCT15 derived from sigmoid colon of the same individual, which is in concordance with the reports demonstrating a decreasing gradient of ABH antigen expression towards the distal part of the colon [40]. However, more research is needed to see if CRC cell line glycosylation is comparable with blood group antigen distribution in the healthy colon. We have also observed that blood group antigen H types 2 and 3 were more abundant than H type 1 antigen in CRC cell lines. Similarly, antibody staining studies showed region-specific expression of mainly H type 1 blood group antigen H in healthy colon tissue, in contrast to colon cancer tissues aberrantly expressing blood group antigens H type 2 and 3 associated with tumour progression in the distal colon and rectum [36].

The majority of the cell line *O*-glycans had α2,3-linked NeuAc at the terminal end of the glycan. Overall sulphation was found to be much less abundant than sialylation, showing the highest expression in SW948 and LS411N cell lines. As compared with normal colon tissue [40], cancer-associated *O*-glycans often show increased sialylation and less sulphation [41]. Furthermore, we detected structures carrying Sd^a^/Cad epitopes only in one cell line, namely, CaCo-2, supporting other reports from the literature [42]. Structures terminated by Sd^a^/Cad epitopes have been described before as characteristic for normal colon mucin tissue [43, 44]. Our results also show a dominance of core 2 and core 1 *O*-linked glycans, and a very low abundance of core 3 and core 4 structures. Core 3 and core 4 are known to be characteristic for the healthy colon mucins [45] and most of the cell lines did not show high expression of those structures. Moreover, core 3 synthase has been reported to be downregulated in colon cancer [46], with a loss of activity in many CRC cell lines [47]. Additionally, a decrease in core 3 structures together with a concomitant increase in core 1 structures has been observed when moving from healthy tissue to tumour regions [15]. Previously, no core 3 structures could be detected in the glycan profiles of five CRC cell lines, in contrast to substantial expression in CRC tissues [27]. We can hypothesize that due to the lack of precursor core 3 structures, *GCNT3*, which is highly expressed in many cell lines, exhibits the core 2 and I-branching activity, resulting in predominance of core 2 and I-branched structures in the cell lines.

To provide a better understanding of regulation of *O*-glycosylation, we also examined the associations of glycan epitopes with transcription factor expression and generated hypotheses by association analysis. *ETS2* transcription factor expression shows associations with I-branched and α2,3-sialylated glycan expression (Fig. 5) supported by its correlation with the expression of the *GCNT3* gene (Supplementary Figure S9)*. ETS2* is a Wnt pathway target gene, whose inactivation leads to increased crypt cell proliferation [48]. It has previously been associated with *MGAT2* promoter activation for the biosynthesis of complex type *N-*glycans; however, its potential regulation of *O-*glycan biosynthesis is still unknown. Oncogenic transcription factor c-Myb, important for both cell proliferation and cell differentiation [49], is also involved in the Wnt pathway activation and shows a positive correlation with the expression of core 1 and α2,6-core sialylated glycans (Fig. 5). Activated β-catenin and *MYB* induce upregulation of *MYC* promoter, and c-Myc transcription factor expression [50]. It has been found that *ST3GAL1, 2 and 4* are transcriptionally upregulated by c-Myc [51], which might contribute to the higher expression of disialyl and sialyl-T antigens, together with a relatively lower expression of core 2 structures (Fig. 5). The very strong negative correlation with blood group antigen H expression in the samples could be explained by the strong correlation of *MYB* gene with *B3GNT3* gene and is in direct competition with the biosynthesis of blood group antigens [31] (Fig. 6). Another strong positive correlation was found between the expression of *ZNF286A* and *ZNF286B* genes and the expression of blood group antigens. This might be a consequence of the strong negative correlations with both the expression of *B3GNT3* and *GCNT3* genes, which are involved in the elongation and branching of *O*-glycan structures. Additionally, a positive correlation with the expression of *ST3GAL3,* encoding the α2,3-sialyltransferase, terminating the elongation of glycan structures with NeuAc residues was found. Further studies need to be performed to confirm these hypotheses.

Although it has been demonstrated before that cell lines can be good representatives of the tumours based on gene mutations, gene expression, and protein expression [6–8], a large-scale study comparing glycosylation of cell lines and tumour tissues is necessary to evaluate whether the cell lines can be used as glycobiological model systems. A small-scale study, which compared *N-* and *O-*glycomes derived from CRC cell lines and tumours, revealed that some cell lines do not represent the *O-*glycosylation of the tumour tissues, showing lack of expression of sialyl-Tn and core 3 disialyl Lewis X hexasaccharide [27], previously associated with malignant transformation [52]. Additionally, we found notable differences between the *O-*glycomes of cell lines cultured at different sites in different culturing conditions; therefore the impact of these variables also needs to be considered.

The presented mass spectrometric approach revealing the complete repertoire of glycans expressed by a specific cell type is a valuable resource for understanding glycosylation-related changes in cancer. While the CRC cell lines have shown enormous diversity of individual glycan structures, their structural features could be associated with the cell differentiation. Moreover, glyco-phenotypes were found to be associated with the expression of specific glycosyltransferases involved in their biosynthesis, providing more insight into the regulation of glycan biosynthesis in different cell types. Finally, this untargeted in-depth screening of cell line glycome phenotypes will provide an important resource for future studies exploring the role of cell glycosylation in CRC progression and drug response leading to discovery of novel targets for the development of anti-cancer antibodies.

## Electronic supplementary material

Below is the link to the electronic supplementary material.

Supplementary file1 (XLSX 1886 kb)

Supplementary file2 (PDF 241 kb)

Supplementary material 1 (.pdf): Supplementary Materials and Methods. Supplementary material 2 (.pdf): Figure S1 Workflow for N- (optional) and O-glycomics from 500,000 cells. The combination and optimization of two previously established protocols [12, 16] allowed sequential release of both N- and O-glycans from cell lysates in a higher throughput manner using 96-well plates. Cell lines were harvested after they were grown up to 80% confluence. Cell lysates from 500,000 cells were used for protein immobilization on PVDF membrane. Proteins were denatured by guanidine HCl and DTT followed by an overnight N-glycan release by PNGase F digestion. After removal of the released N-glycans, O-glycans were released by reductive β-elimination, purified by cation exchange chromatography (CEX) and graphitized carbon solid phase extraction (SPE) packed in a 96-well filter plates and finally analysed by PGC nano-LC-ESI-MS/MS. Supplementary material 2(.pdf): Figure S2 Technical and biological variation. PC1 and PC2 score plot (a) together with PC2 and PC3 score plot (b) based on monosaccharide average compositions of all technical (marked with 1 and 2) and biological replicates (marked with a, b and c) of cell lines and fetuin O-glycan standard (control) display close clustering of scores showing robustness of the method. The top three principal components explain 86.85% of the variation within the data. Cell lines cultured at VUMC are marked with “VU”. Supplementary material 2(.pdf): Figure S3 Structural diversity of CRC cell line O-glycans. Relative abundances of individual O-linked glycans released from 26 CRC cell lines. Glycans are numbered according to Supplementary Table 2: S1. The most abundant O-glycans in different cell lines are displayed in the legend. Sulphate modification is indicated with “S”. Reduced reducing end is indicated by a circle on the reducing end of the glycan. Different classifications of the cell lines are displayed with colour codes based on gene expression (colon-like in red, undifferentiated in light blue, and non-assigned in grey). Supplementary material 2(.pdf): Figure S4 Abundance of structural glycan features per cell type. Geometrical tile of the rescaled relative abundance (%) of the calculated structural glycan features (x-axis) and cell line type (y-axis). Different classification of the cell lines is displayed with colour code based on gene expression (colon-like in red and undifferentiated in light blue) and consensus molecular subtypes (CMS1 in yellow, CMS2 in dark blue, CMS3 in pink and CMS4 in green). Non assigned cell lines were marked in grey for the gene expression as well as CMS status. All variables have been rescaled fitting within a 0 up to 1 scale. Supplementary material 2(.pdf): Figure S5 Glycan structure determination of major fucosylated glycan structures from LS180 cell line. (a) In order to reveal structural information about all three isomers at m/z 1186.40 in the sample α2,3 Neuraminidase digestion was performed. (b) Digestion resulted in a loss of two peaks (RT= 30 and 32 min) carrying α2,3-linked sialic acid(s), and one remaining isomer Gal(Fuc)GlcNAcβ1-3Galβ1-3(NeuAcα2-6)GalNAcol (RT= 23 min). (a) and (b) The loss of two isomers resulted a significant increase in the abundance of the neutral isomer Galβ1-3 [Gal(Fuc)GlcNAcβ1-6]GalNAcol upon digestion. (c) The terminal Lewis epitope was confirmed by a characteristic Z/Z ion at m/z 551.23 and Z/Z-CH2O at m/z 521.22. (d) The structure of the remaining isomer at m/z 1186.40 (RT= 23 min) was further confirmed by the fragmentation spectra. Supplementary material 2(.pdf): Figure S6 Glycan linkage determination of Lewis epitopes expressed by LS180 cell line. (a) Additional enzymatic cleavage of the α2,3 neuraminidase digested sample was performed with combined α1,3/4 fucosidase and β1,4 galactosidase digestion in order to distinguish between (sialyl) Lewis x and a epitopes. (a) A significant increase in intensity of the remaining structures Galβ1-3(GlcNAcβ1-6)GalNAcol at m/z 587.30 and GlcNAcβ1-3Galβ1-3(NeuAcα2-6)GalNAcol at m/z 878.32 compared to control (b) suggest the presence of terminal sialyl-Lewis x epitope Galβ1-4(Fucα1-3)GlcNAc-R on the isomer eluting at RT=34 min. (d) The fragmentation of GlcNAcβ1-3Galβ1-3(NeuAcα2-6)GalNAcol revealed the diagnostic ion at m/z 495.21 for the α2,6-linked sialic acid to the reducing end GalNAc structures. Supplementary material 2(.pdf): Figure S7 Glycan linkage determination of major fucosylated glycan structures from CaCo-2 cell line. (a) The structures of two isomers present at m/z 1186.40 in the sample could be determined from the fragmentation spectra of the sialylated species, by the presence of the diagnostic fragment ion at 0,2A3α-H2O at m/z 409.08 (b), confirming the blood group antigen type 2 in the isomer eluting at 52.4 min. (c) The 0,4A0α fragment at m/z 424.04 of the reducing end GalNAc revealed the composition of the 6 arm of the core 2 isomer at RT 63.8 min, together with the Y1α ion at m/z 530.25 indicating the blood group antigen type 3 on the 3 arm. Supplementary material 2(.pdf): Figure S8 Supplementary figure S8 Differentially expressed glycosyltransferase and transcription factor genes in colon like versus undifferentiated cell lines. Volcano plot displays a selection of genes involved in O-glycan biosynthesis (depicted by circles) and transcription factors (depicted by triangles) which show the highest fold change in expression (loge > 0.5) when comparing colon-like and undifferentiated cell lines with high statistical significance (Bonferroni corrected p-value < 0.05). Genes upregulated in colon-like cell lines are marked in red, while genes upregulated in undifferentiated cell lines are marked in blue [8]. The genes that do not pass the thresholds are marked in grey. Supplementary material 2(.pdf): Figure S9 Correlation between the expression of glycosyltransferase encoding genes and transcription factors. A Spearman’s nonparametric correlation was performed with significance α=0.05. Crosses indicate non statistically significant correlations [8] (PDF 2746 kb)

Supplementary Table 1 (.xlsx): S1- Calculated monosaccharide average compositions (MSAC) per technical (1 or 2) or biological replicate (A, B or C). The MSAC represents the normalized number of sugar residues and modifications per glycan molecule (hexose, N-acetylhexosamine, sialylation, sulphation, fucosylation). The intensities of corresponding structures were multiplied by the monosaccharide compositions and summed over the entire sample. N: N-acetylhexosamine, H: hexose, F: fucose, S: N-acetylneuraminic acid, Su: sulphate residue. Supplementary Table 1: S2-S31 (.xlsx) - O-linked glycans present in fetuin standard (S2), and different cell lines (S3-S31) with their retention times (min), peak areas, relative abundances with standard deviation and relative standard deviation (CV), and calculations of monosaccharide average compositions for each technical (1-3) replicate (XLSX 372 kb)

Supplementary Table 2 (.xlsx): S1 - Overview of individual O-linked glycans present in colorectal cancer cell lines, with their retention time (min), relative abundances per cell line, standard deviation and relative standard deviation (CV). The glycan relative abundances for each cell line were averaged for two technical and 3 biological replicates (SW480, SW620, HCT8, HCT116, HT29). Identification of glycans was performed based on PGC retention time, known biosynthetic pathways and manual inspection of fragmentation spectra following known MS/MS fragmentation pathways of O-glycan alditols in negative-ion mode. Glycan sequences and linkages were confirmed by the analysis of neutral glycans upon α2-3 neuraminidase digestion. Annotated glycan structures were used for calculations of structural glycan features calculated by summing relative intensities of each glycan multiplied by the number of epitopes per glycan. This was performed for the structures which could be unambiguously annotated. Blue square: N-acetylglucosamine, yellow circle: galactose, red triangle: fucose, pink diamond: N-acetylneuraminic acid, N: N-acetylhexosamine, H: hexose, F: fucose, S: N-acetylneuraminic acid, Su: sulphate residue. Supplementary Table 2: S2 (.xlsx) - Characteristics of cell lines used in this study together with their calculated structural glycan features. Information about the cell lines was obtained from ATCC and referenced literature. Structural glycan features were calculated and averaged per cell type (XLSX 3311 kb)

## Data Availability

The raw mass spectrometric data files that support the findings of this study are available in GlycoPOST in mzXML format, with the identifier GPST000035, accessible via the following link https://glycopost.glycosmos.org/preview/863090025d7bddcbd8bcf. The MS/MS spectra of glycan structures are available in UnicarbDR repository, accessible via the following link https://unicarb-dr.biomedicine.gu.se/references.

## Abbreviations

CDX1: Caudal type homeobox 1
CMS: Consensus molecular subtype
CRC: Colorectal cancer
CRCSC: Colorectal Cancer Subtyping Consortium
DTT: Dithiothreitol
EMT: Epithelial to mesenchymal transition
GalNAc: *N*-acetylgalactosamine
GlcNAc: *N*-acetylglucosamine
GT: Glycosyltransferase
KOH: Potassium hydroxide
MeCN: Acetonitrile
MeOH: Methanol
MSAC: Mass spectrometry average composition
MSI: Microsatellite instability
NaBH4: Sodium borohydride
NeuAc: *N*-acetylneuraminic acid
NK: Natural killer
PCA: Principal component analysis
PGC nano-LC–ESI-MS/MS: Porous graphitized carbon nano-liquid chromatography electrospray mass spectrometry
PVDF: Polyvinylidene difluoride
TFA: Trifluoroacetic acid
TGF: Transforming growth factor
