# Supplementary material for: Colorectal cancer cell lines show striking diversity of their O-glycome reflecting the cellular differentiation phenotype
Source: Cell Mol Life Sci. 2020 Mar 31;78(1):337–50. doi: 10.1007/s00018-020-03504-z (PMC7867528; doi:10.1007/s00018-020-03504-z)
Supplement: Supplementary file 2 — Supplementary file2 (PDF 241 kb) [file 18_2020_3504_MOESM2_ESM.pdf]

Supplementary material 1

Colorectal cancer cell lines show striking diversity of their O-glycome reflecting the cellular differentiation phenotype

Katarina Madunic[1], Tao Zhang[1], Oleg A. Mayboroda[1], Stephanie Holst[1], Kathrin Stavenhagen[1], Chunsheng Jin[2], Niclas G. Karlsson[2], Guinevere S.M. Lageveen-Kammeijer[1], Manfred Wuhrer[1,*]

[1] *Leiden University Medical Center, Center for Proteomics and Metabolomics, Postbus 9600, 2300 RC Leiden, The Netherlands*

[2] *Department of Medical Biochemistry and Cell Biology, Institute of Biomedicine, Sahlgrenska Academy, University of Gothenburg, Gothenburg, Sweden*

**Corresponding author: m.wuhrer@lumc.nl*

SUPPLEMENTARY MATERIALS AND METHODS

MATERIALS AND REAGENTS

Trifluoroacetic acid (TFA; cat. nr. 1.38178.0050), fetuin from fetal bovine serum (cat. nr. F3385; lot nr. 078K74351), tris(hydroxymethyl)amino-methane (cat. nr. 252859; lot#BCBM2559V), sodium borohydride ($NaBH_4$; cat. nr. 452882; lot nr. STBD8912V), hydrochloric acid (HCl; cat. nr. 258148; lot nr. SZBD3100V), DL-Dithiothreitol (DTT; cat. nr. D0632; lot nr. SLBW0160), 8 M guanidine hydrochloride (GuHCl; cat. nr. 24115; lot nr. QE216133), ammonium bicarbonate (cat. nr. 09830; lot nr. BCBQ6426V,) cation exchange resin Dowex 50W X8 (cat. nr. 217492; lot nr. MKCH2513), ammonium acetate (cat. nr. A1542), and α1-3,4-fucosidase solution from *Xanthomonas* sp. (cat. nr. F3023-1VL, lot nr. SLBM4470V) together with its reaction buffer (cat. nr. E5879-1VL) were purchased from Sigma Aldrich (St. Louis, MO); ethanol (cat. nr. 100983.1000), sodium chloride (NaCl ; cat. nr. 1.06404.1000), and methanol (MeOH; cat. nr. 1.06009.2500) were purchased from Merck (Darmstadt, Germany). Acetonitrile LC-MS grade (MeCN; cat. nr. 01203502) was obtained from Biosolve (Valkenswaard, The Netherlands), Ethylenediaminetetraacetic acid solution pH 8.0 (cat. nr. 03690; lot nr. BCBL9265V), glacial acetic acid (cat. nr. A6283; lot nr. SZBG2660H), and potassium hydroxide (KOH; cat. nr. P1767) from Honeywell Fluka (Charlotte, North Carolina, USA), PNGase F (Flavobacterium meningosepticum recombinant in E. coli; Cat No. 11365193001) and complete EDTA free protease inhibitor cocktail tablets (cat. nr. 05892791001) from Roche (Mannheim, Germany), SPE bulk sorbent Carbograph (cat. nr. 1769; lot nr. 5122145) from Grace discovery

sciences (Columbia, TN, USA), MultiScreen® HTS 96-multiwell plates (pore size 0.45m) with high protein-binding membrane (hydrophobic Immobilon-P PVDF membrane) and 96-well PP Microplate (cat. nr. 651201; lot nr. E1708385) were purchased from Millipore (Amsterdam, The Netherlands), 96-well PP filter plate (cat. nr. OF1100) from Orochem technologies (Naperville, Illinois, USA), α2-3 neuraminidase (cat. nr.. GK80040), β1-4 galactosidase S (cat. nr. P0745L, lot nr. 0011606) and Glycobuffer 1 (cat. nr. B1727S) from New England Biolabs (Ipswich, MA, USA), Hepes-buffered RPMI 1640 (cat. nr. 52400-025) and Dulbecco's Modified Eagle (DMEM) culture media (cat. nr. 670087) were purchased from Gibco (Paisley, UK), fetal bovine serum (FBS) (cat. nr. 26140079) and penicillin/streptomycin (cat. nr. 15070063) at Invitrogen (Carlsbad, California, USA), 0.5% trypsin-EDTA solution 10X (cat.nr. sc-363354) was obtained from Santa Cruz Biotechnology (Dallas, Texas, USA), and T75 cell culture flasks (cat. nr. 658170) from Greiner-Bio One B.V. (Alphen aan de Rijn, The Netherlands). All buffers were prepared using Milli-Q water (mQ) generated from a Q-Gard 2 system (Millipore, Amsterdam, Netherlands), maintained at ≥ 18 MΩ.

CELLS AND CELL CULTURE

Human CRC cell lines were obtained from the Department of Surgery of the Leiden University Medical Center (LUMC), Leiden, The Netherlands, as well as the Department of Pathology of the VU University Medical Center (VUmc), Amsterdam, The Netherlands. The cell lines cultured at the LUMC were kept in Hepes-buffered RPMI 1640 culture medium containing L-glutamine and supplemented with penicillin (5000 IU per mL), streptomycin (5 mg/ml), and 10% (v/v) FBS. Cells at the VuMC were cultured in DMEM medium, supplemented with 10% (v/v) FBS and antibiotics, except for the KM12 cell line, which was grown in RPMI/10% FCS/antibiotics and L-glutamine at the VUmc. Cells were incubated at 37˚C with 5% CO_2 in humidified air. The cells were harvested after reaching 80% of confluence. To detach the cells from the culture flask a trypsin/EDTA solution in 1X PBS was used. Enzyme activity was stopped using the medium in a ratio 2:5 (trypsin:medium *v/v*). The cells were counted using TC20 automated cell counter from Bio-Rad technologies (Hercules, California, USA) based on trypan blue staining. The cells were washed twice with 5 mL of 1x PBS, aliquoted to 2.0 x 10^6 cells per mL of 1x PBS and pelleted by centrifuging 3 min at 1500 x *g*. Finally, the supernatant was removed, and the cell pellets were stored at -20˚C.

O-GLYCAN RELEASE

Cell pellets containing 2 million cells were resuspended in 100 µL of lysis buffer and sonicated for 60 min in an ultrasonic bath at 60°C. Meanwhile, the 96-well plates with hydrophobic Immobilon-P PVDF membrane were preconditioned with 1x 100 µL of 70 % ethanol and 2x with 100 µL of water. Cell lysate suspensions (25 µL)

containing 500,000 cells were loaded to the PVDF membrane wells in triplicates, and mixed with 75 µL of denaturation mix containing 5.8 M GuHCl and 5 mM DTT. The plate was incubated in a humidified plastic box for 60 min at 60°C. After incubation, the controls containing 20 µg of bovine fetuin and 10 µg porcine stomach mucin were loaded onto the PVDF wells. Denaturation agents were removed by washing three times with MQ water followed by centrifugation at 500 x *g* for 1-2 min. A PNGase F (Roche) mix (15 µL) was added to each well containing 2 µL of enzyme and 13 µL of MQ. The plate was incubated for 15 min at 37˚C. Additionally, 15 µL of water was added in each well. The plate was incubated overnight in a humidified plastic box at 37˚C. Released *N*-glycans were recovered from the PVDF plate by centrifugation and washing three times with 30 µL of water and stored at -20˚C. The analysis of the released *N*-glycans was not in the scope of this study. For the release and reduction of *O*-glycans, 50 µL of 0.5 M $NaBH_4$ in 50 mM KOH was added to each well, and incubated for 16 hours at 50˚C in a humidified plastic box to minimise evaporation. The released *O*-glycans were recovered by centrifugation and washing 3x with 30 µL of water. To neutralize the reaction, 3 µL of glacial acetic acid was added to each sample. Desalting of the samples was performed using a strongly acidic cation exchange resin which was packed into 96-well filter plates. Briefly, 100 µL of the resin suspension in MeOH (50/50, v/v) was added to each well in the filter plate. The columns were preconditioned by 3x 100 µl of 1M HCl, followed by 3x 100 µL MeOH and 3x 100 µL water each time removed using the vacuum manifold. The samples containing *O*-glycans were loaded onto the columns and eluted two times with 40 µL of water followed by centrifugation at 500 x *g*. The samples were dried in a Speed Vac concentrator at 30˚C. The remaining borate was removed by several rounds of co-evaporation using 100 µL MeOH in the SpeedVac concentrator. Solid phase extraction cleaning step was performed by packing 50 µL of bulk sorbent carbograph slurry in MeOH (approximate concentration 50/50, v/v) into 96-well filter plates. The columns were preconditioned by 3x 100 µL wash with 80% MeCN 0.1% TFA, and 3x 100 µL 0.1% TFA. Upon sample loading the columns were washed twice with 40 µL of 0.1% TFA, followed by *O*-glycan elution by 3x 40 µL of 60% MeCN 0.1% TFA. Samples were dried in a SpeedVac concentrator and stored at -20˚C freezer.

EXOGLYCOSIDASE DIGESTIONS

As negative mode MS/MS spectra of highly sialylated glycan species does not provide enough informative fragment ions, the third technical replicate of all prepared cell line samples was subjected to α2-3 neuraminidase digestion, and fragmentation spectra of neutral glycan species was used for further detailed structural elucidation. Briefly, each sample was resuspended in 18 µL of Glycobuffer 1 (New England Biolabs, Ipswich, MA, USA), and

split into two aliquots, one for digestion and the other one as a control. Two µL of α2-3 neuraminidase was added to the samples for digestion, and 2 µL of Glycobuffer 1 to the controls.

LS180 cell line *O*-glycans were prepared again to perform additional exoglycosidase digestions following as described in the *O*-glycan release subsection. Briefly, the released *O*-glycans were resuspended in 10 µL of milliQ water, 2 µl of reaction buffer containing 50 mM sodium phosphate (pH 5.0) was added, and 2 µL of α1-3,4-fucosidase together with 2 µl of β1-4 galactosidase was added to the reaction vial. The samples were incubated overnight at 37˚C and cleaned by PGC SPE, as described earlier [16].

ANALYSIS OF RELEASED N- AND O-GLYCANS USING PGC-LC-ESI-MS/MS

Analysis was performed using a PGC nano-LC Ultimate 3000 UHPLC system (Dionex/Thermo, Sunnyvale, California, USA) coupled to an amaZon ETD speed ion trap (Bruker, Bremen, Germany). The samples were re-dissolved in 12 µL of water, and 4 µL of each sample was injected into the system. Hypercarb™ KAPPA 30 × 0.32 mm, 5 µm particle size trap column was used to load the samples using 98% buffer A (10 mM ammonium bicarbonate) at a loading flow of 6 µL min^{-1}. The equilibration conditions for the separation column were 2% buffer B (60% MeCN in 10 mM ammonium bicarbonate). The reduced *O*-glycans were separated on a Hypercarb PGC Column, Thermo, 100 mm × 75 µm, 3 µm particle size at a 0.6 µL min^{-1} flow rate by applying a linear gradient from 2% to 50% buffer B over 73 min. The column was held at a constant temperature of 45˚C. The LC system was coupled to an amaZon ETD speed ESI ion trap MS using the CaptiveSpray™ source (Bruker) with an applied capillary voltage of 1000 V and a dry gas temperature of 280˚C at 5 L min^{-1} and nebulizer at 5 psi. MeCN enriched dopant gas was used for increasing sensitivity (Bruker NanoBooster). Deprotonated ions were generated by negative ionization mode. MS spectra were acquired within a mass to charge ratio (*m/z*) range of 380-1850 in enhanced mode, target mass of smart parameter setting was set to *m/z* 900; ion charge control (ICC) to 40,000 and maximum acquisition time to 200 ms. MS/MS spectra were generated using collision induced dissociation over an *m/z* range from 100-2500 on the four most abundant precursors, applying an isolation width of *m/z* 3. The fragmentation cut-off was set to 27% with 100% fragmentation amplitude using the Enhanced SmartFrag option from 30-120% in 32 ms and ICC was set to 150,000.

DATA PROCESSING

MS spectra were manually screened for *O*-glycan compositions, with help of Glycoworkbench [2] and Glycomod [3] software. Identification of glycans was performed based on PGC retention time, known biosynthetic pathways, and manual inspection of fragmentation spectra following known MS/MS fragmentation pathways of *O*-glycan

alditols in negative-ion mode [4, 5] Glycan sequences and linkages were confirmed by the analysis of glycans upon α2-3 neuraminidase digestion. Extracted ion chromatograms were used to integrate area under the curve (AUC) for each individual glycan isomer using Compass DataAnalysis software v.5.0. Peaks were manually picked and integrated if the signal to noise ratio ≥ 6 in both technical and biological replicates. Selected glycan peaks were confirmed by MS/MS. Relative quantitation was performed on the total area of all *O*-glycans within one sample normalizing it to 100%. Glycan epitopes were grouped by summing relative intensities of each glycan multiplied by the number of epitopes per glycan. This was performed for the structures which could be unambiguously annotated. We have identified glycans representing more than 90% of the relative intensity for each cell line. For 13 cell lines, we have identified glycans representing more than 95% of the relative intensity per cell line. MS/MS mass lists were exported from the DataAnalysis software and stored in Glycoworkbench workspace prior to uploading to Unicarb DR repository [6]. Selection of genes of interest from the CRC cell line transcriptomics dataset [7] was performed using a cut off p-value < 0.05 (Bonferroni corrected) and fold change $\log_e > 0.5$ when comparing colon-like and undifferentiated cell lines.

STATISTICS

Due to the very high variability in the expression of individual glycan structures between the cell lines, they were grouped into calculated structural glycan features. An imputation of the minimum positive number (0.0001) was performed to enable use of the statistical tools sensitive to the missing values such as principal component analysis. Data analysis and visualization was performed in ''R'' software, version 3.5.1, using packages "pcaMethods", ''Rcpm'', "ggplot2", and ''Tidyverse2''. Regularized canonical correlation analysis was performed using rcc function as it is implemented in the "mixOmics" package [8]. For the result visualization a Clustered Image Map-CIM (cim function of the "mixOmics" package) was used. In the CIM display, each coloured block represents an association subsets of the glycan epitope variables and the transcript expression variables. Numerical values for the associations are given as "r" in the results section.

REFERENCES

1. Jensen PH, Karlsson NG, Kolarich D, Packer NH (2012) Structural analysis of N- and O-glycans released from glycoproteins. Nat Protoc 7:1299–1310. https://doi.org/10.1038/nprot.2012.063

2. Ceroni A, Maass K, Geyer H, et al (2008) GlycoWorkbench: A tool for the computer-assisted annotation of mass spectra of glycans. J Proteome Res 7:1650–1659. https://doi.org/10.1021/pr7008252

3. Cooper CA, Gasteiger E, Packer NH (2001) GlycoMod - A software tool for determining glycosylation compositions from mass spectrometric data. Proteomics 1:340–349. https://doi.org/10.1002/1615-9861(200102)1:2<340::AID-PROT340>3.0.CO;2-B

4. Karlsson NG, Schulz BL, Packer NH (2004) Structural determination of neutral O-linked

oligosaccharide alditols by negative ion LC-electrospray-MSn. J Am Soc Mass Spectrom 15:659–672. https://doi.org/10.1016/j.jasms.2004.01.002

5. Karlsson NG, Wilson NL, Wirth HJ, et al (2004) Negative ion graphitised carbon nano-liquid chromatography/mass spectrometry increases sensitivity for glycoprotein oligosaccharide analysis. Rapid Commun Mass Spectrom 18:2282–2292. https://doi.org/10.1002/rcm.1626

6. Rojas-Macias MA, Mariethoz J, Andersson P, et al (2019) Towards a standardized bioinformatics infrastructure for N- and O-glycomics. Nat Commun 10:3275. https://doi.org/10.1038/s41467-019-11131-x

7. Berg KCG, Eide PW, Eilertsen IA, et al (2017) Multi-omics of 34 colorectal cancer cell lines - a resource for biomedical studies. Mol Cancer 16:116. https://doi.org/10.1186/s12943-017-0691-y

8. Rohart F, Gautier B, Singh A, Lê Cao K-A (2017) mixOmics: An R package for ’omics feature selection and multiple data integration. PLoS Comput Biol 13:e1005752. https://doi.org/10.1371/journal.pcbi.1005752
