# Supplementary material for: Colorectal cancer cell lines show striking diversity of their O-glycome reflecting the cellular differentiation phenotype
Source: Cell Mol Life Sci. 2020 Mar 31;78(1):337–50. doi: 10.1007/s00018-020-03504-z (PMC7867528; doi:10.1007/s00018-020-03504-z)
Supplement: Supplementary file 3 — Supplementary material 1 (.pdf): Supplementary Materials and Methods. Supplementary material 2 (.pdf): Figure S1 Workflow for N- (optional) and O-glycomics from 500,000 cells. The combination and optimization of two previously established protocols [12, 16] allowed sequential release of both N- and O-glycans from cell lysates in a higher throughput manner using 96-well plates. Cell lines were harvested after they were grown up to 80% confluence. Cell lysates from 500,000 cells were used for protein immobilization on PVDF membrane. Proteins were denatured by guanidine HCl and DTT followed by an overnight N-glycan release by PNGase F digestion. After removal of the released N-glycans, O-glycans were released by reductive β-elimination, purified by cation exchange chromatography (CEX) and graphitized carbon solid phase extraction (SPE) packed in a 96-well filter plates and finally analysed by PGC nano-LC-ESI-MS/MS. Supplementary material 2(.pdf): Figure S2 Technical and biological variation. PC1 and PC2 score plot (a) together with PC2 and PC3 score plot (b) based on monosaccharide average compositions of all technical (marked with 1 and 2) and biological replicates (marked with a, b and c) of cell lines and fetuin O-glycan standard (control) display close clustering of scores showing robustness of the method. The top three principal components explain 86.85% of the variation within the data. Cell lines cultured at VUMC are marked with “VU”. Supplementary material 2(.pdf): Figure S3 Structural diversity of CRC cell line O-glycans. Relative abundances of individual O-linked glycans released from 26 CRC cell lines. Glycans are numbered according to Supplementary Table 2: S1. The most abundant O-glycans in different cell lines are displayed in the legend. Sulphate modification is indicated with “S”. Reduced reducing end is indicated by a circle on the reducing end of the glycan. Different classifications of the cell lines are displayed with colour codes based on gene expr [file 18_2020_3504_MOESM3_ESM.pdf]

Supplementary material

Colorectal cancer cell lines show striking diversity of their O-glycome reflecting the cellular differentiation phenotype

Katarina Madunic[1], Tao Zhang[1], Oleg A. Mayboroda[1], Stephanie Holst[1], Kathrin Stavenhagen[1], Chunsheng Jin[2], Niclas G. Karlsson[2], Guinevere S.M. Lageveen-Kammeijer[1], Manfred Wuhrer[1,*]

[1] *Leiden University Medical Center, Center for Proteomics and Metabolomics, Postbus 9600, 2300 RC Leiden, The Netherlands*

[2] *Department of Medical Biochemistry and Cell Biology, Institute of Biomedicine, Sahlgrenska Academy, University of Gothenburg, Gothenburg, Sweden*

**Corresponding author: m.wuhrer@lumc.nl*

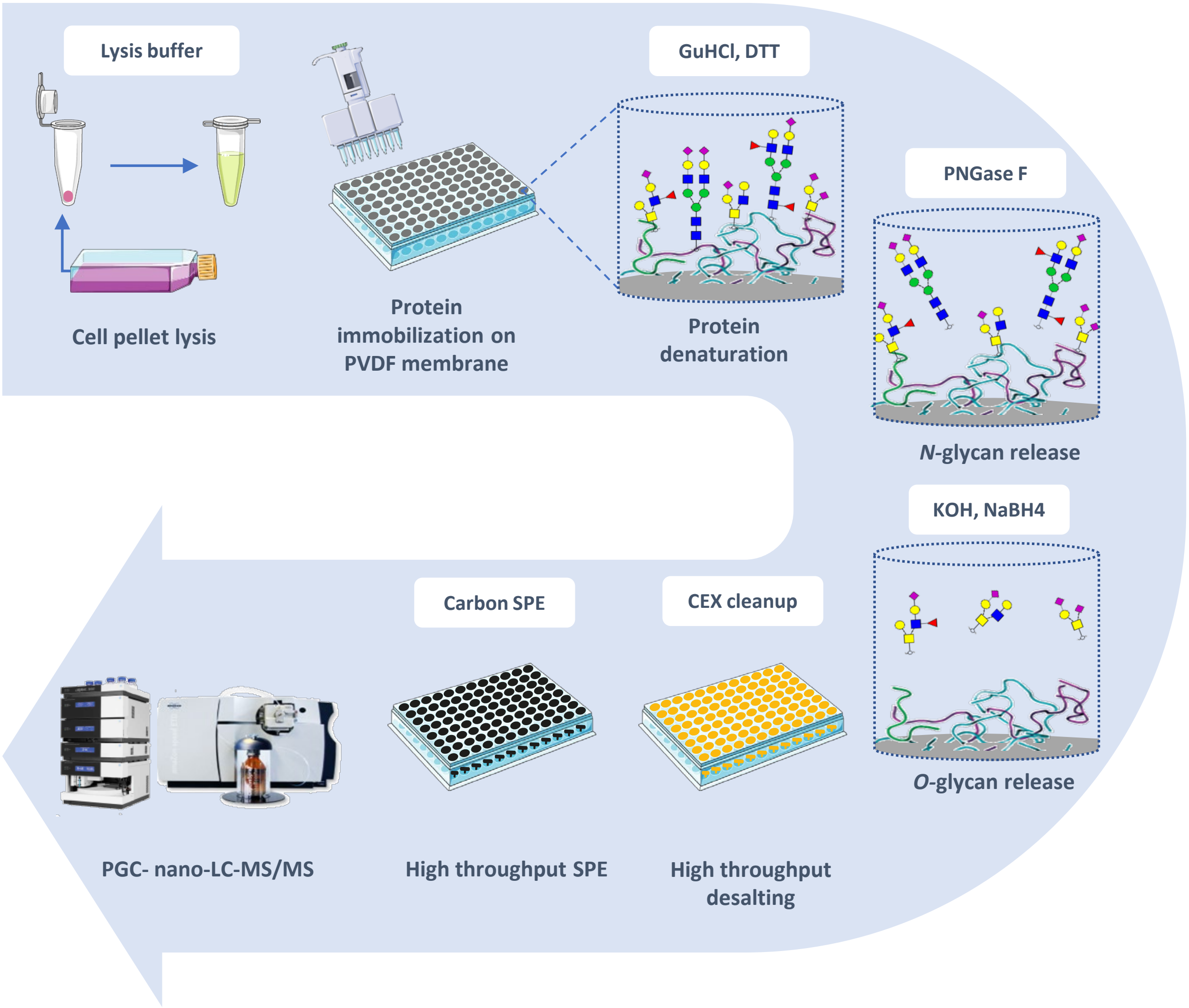

Supplementary figure S1 Workflow for *N*- (optional) and *O*-glycomics from 500,000 cells. Cell lines were harvested after they were grown up to 80% confluence. Cell lysates from 500,000 cells were used for protein immobilization on PVDF membrane. Proteins were denatured by guanidine HCl and DTT followed by an overnight *N*-glycan release by PNGase F digestion. After removal of the released *N*-glycans, *O*-glycans were released by reductive β-elimination, purified by cation exchange chromatography (CEX) and graphitized carbon solid phase extraction (SPE) packed in a 96-well filter plates and finally analyzed by PGC nano-LC-ESI-MS/MS

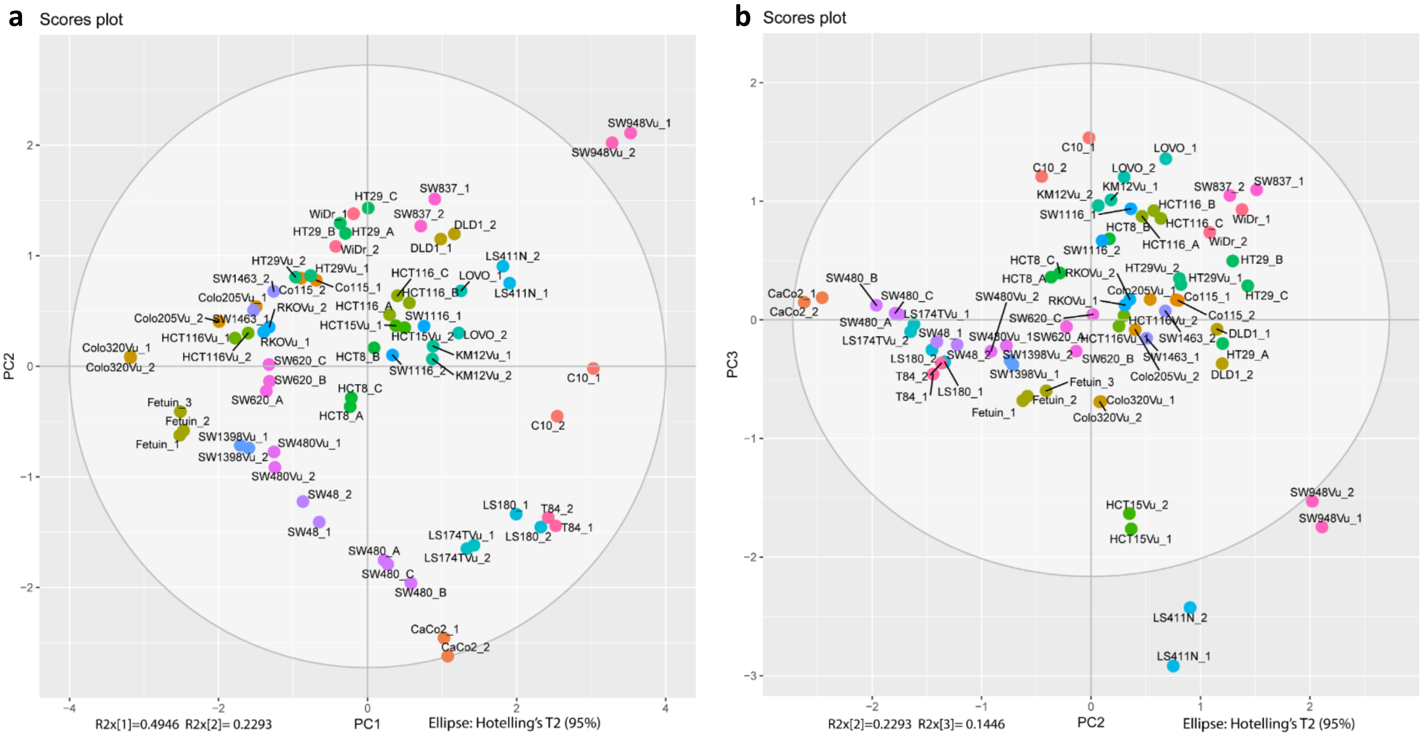

Supplementary figure S2 Technical and biological variation. PC1 and PC2 score plot (**a**) together with PC2 and PC3 score plot (**b**) based on monosaccharide average compositions of all technical (marked with 1 and 2) and biological replicates (marked with a, b and c) of cell lines and fetuin *O*-glycan standard (control) display close clustering of scores showing robustness of the method. The top three principal components explain 86.85% of the variation within the data. Cell lines cultured at VUMC are marked with “VU”.

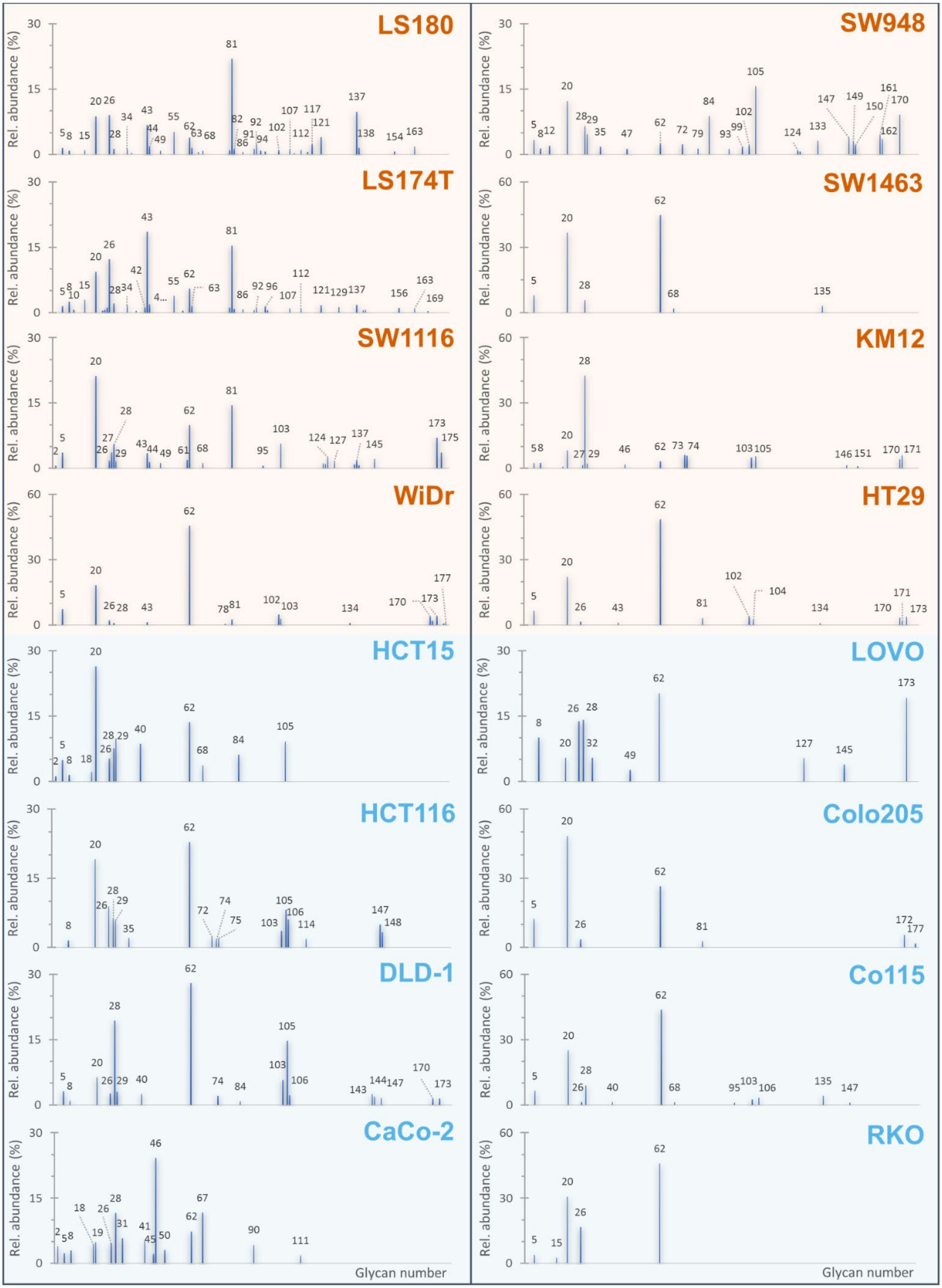

Supplementary figure S3

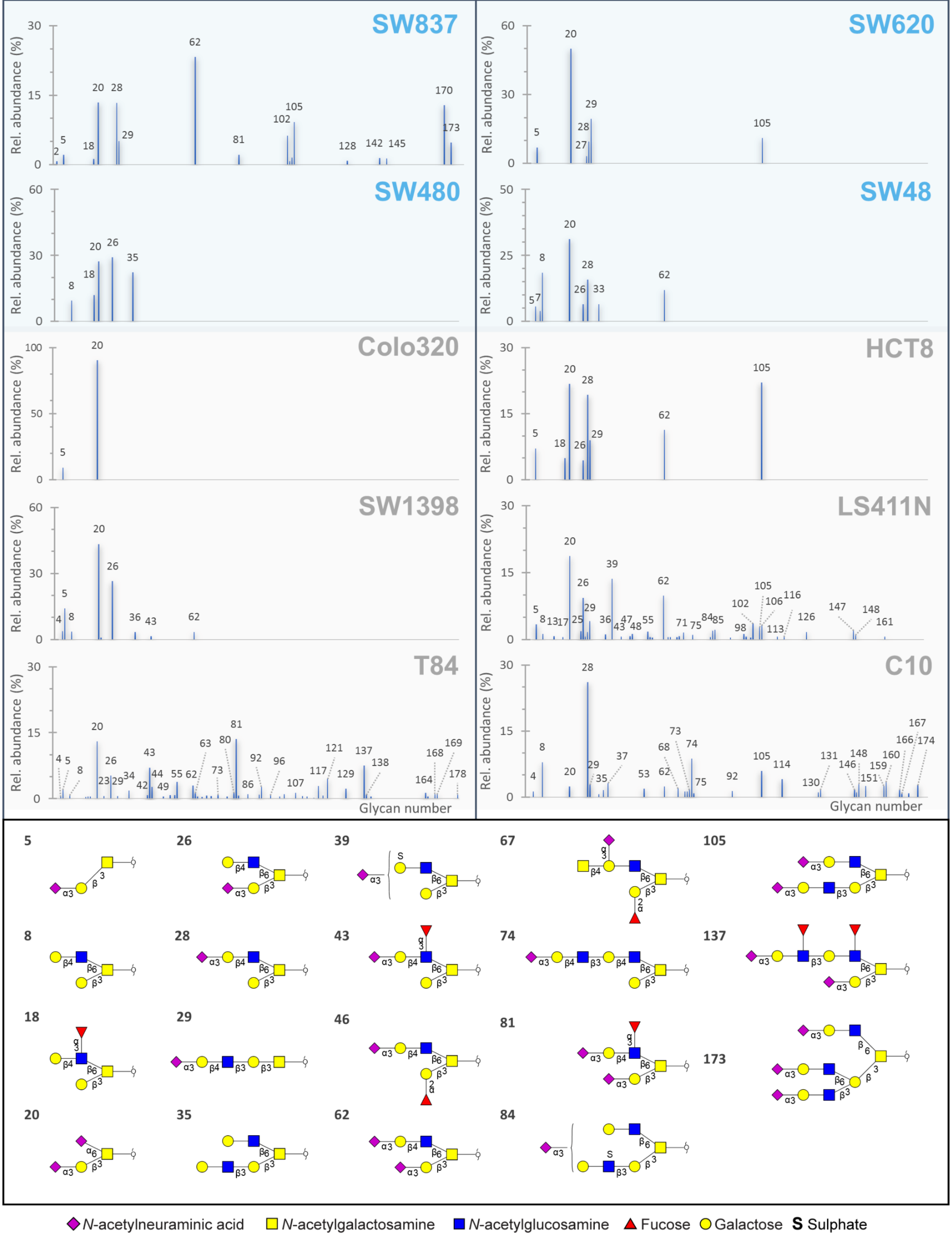

Supplementary figure S3 Structural diversity of CRC cell line *O*-glycans. Relative abundances of individual *O*-linked glycans released from 26 CRC cell lines. Glycans are numbered according to Additional file 3: Table S1. The most abundant *O*-glycans in different cell lines are displayed in the legend. Sulphate modification is indicated with “S”. Reduced reducing end is indicated by a circle on the reducing end of the glycan. Different classifications of the cell lines are displayed with color codes based on gene expression (colon-like in red, undifferentiated in light blue, and non-classified in gray).

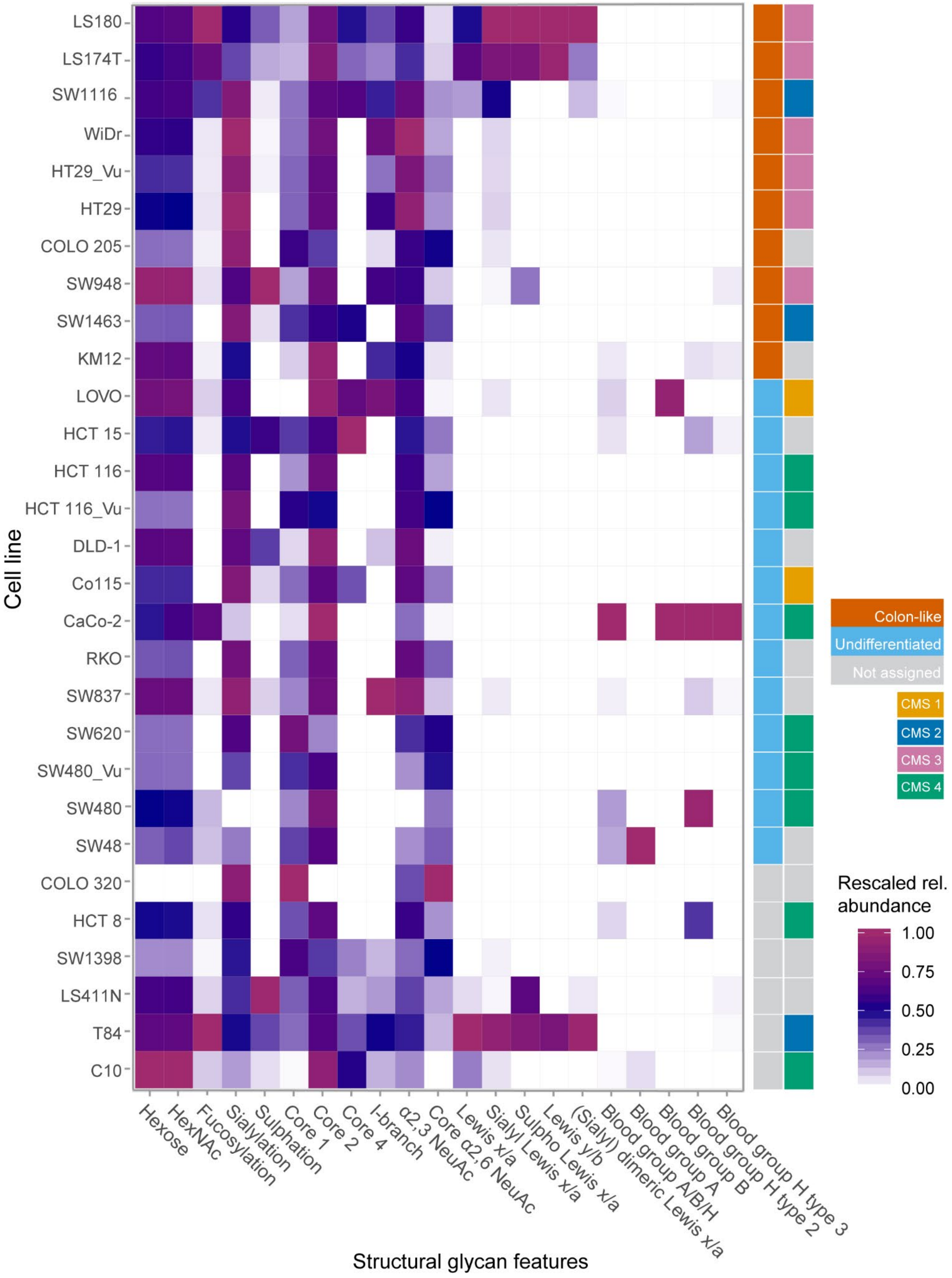

Supplementary figure S4 Abundance of structural glycan features per cell type. Geometrical tile of the rescaled relative abundance (%) of the calculated structural glycan features (x-axis) and cell line type (y-axis). Different classification of the cell lines is displayed with color code based on gene expression (colon-like in red and undifferentiated in light blue) and consensus molecular subtypes (CMS1 in yellow; CMS2 in dark blue; CMS3 in pink and CMS4 in green). Non classified cell lines were marked in gray for the gene expression as well as CMS status. All variables have been rescaled fitting within a 0 up to 1 scale.

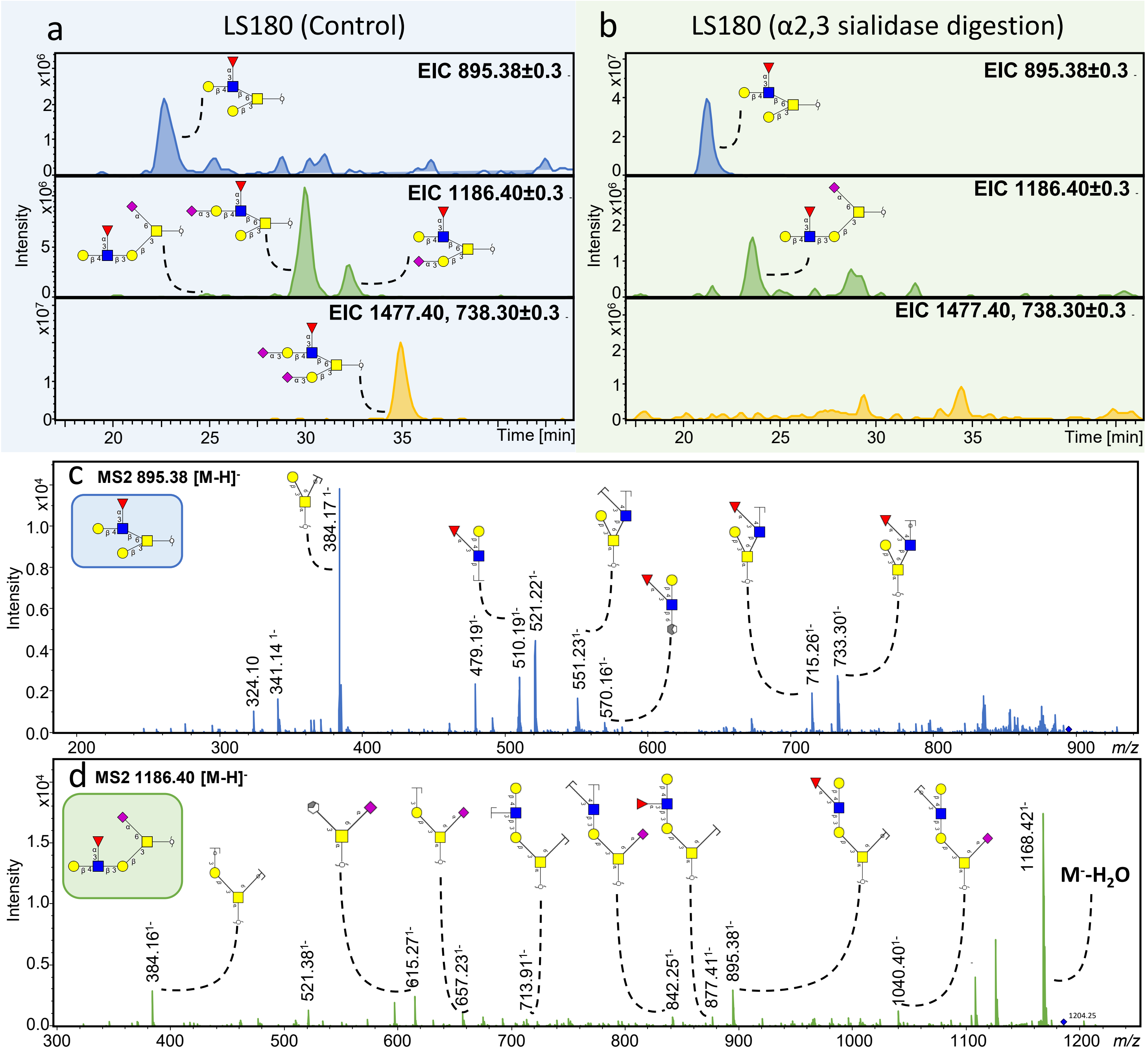

Supplementary figure S5 Glycan structure determination of major fucosylated glycan structures from LS180 cell line. (**a**) In order to reveal structural information about all three isomers at *m/z* 1186.40 in the sample α2,3 Neuraminidase digestion was performed. (**b**) Digestion resulted in a loss of two peaks (RT= 30 and 32 min) carrying α2,3-linked sialic acid(s), and one remaining isomer Gal(Fuc)GlcNAcβ1-3Galβ1-3(NeuAcα2-6)GalNAcol (RT= 23 min). (**a**) and (**b**) The loss of two isomers resulted a significant increase in the abundance of the neutral isomer Galβ1-3[Gal(Fuc)GlcNAcβ1-6]GalNAcol upon digestion. (**c**) The terminal Lewis epitope was confirmed by a characteristic Z/Z ion at *m/z* 551.23 and Z/Z-CH_2O at *m/z* 521.22. (**d**) The structure of the remaining isomer at *m/z* 1186.40 (RT= 23 min) was further confirmed by the fragmentation spectra.

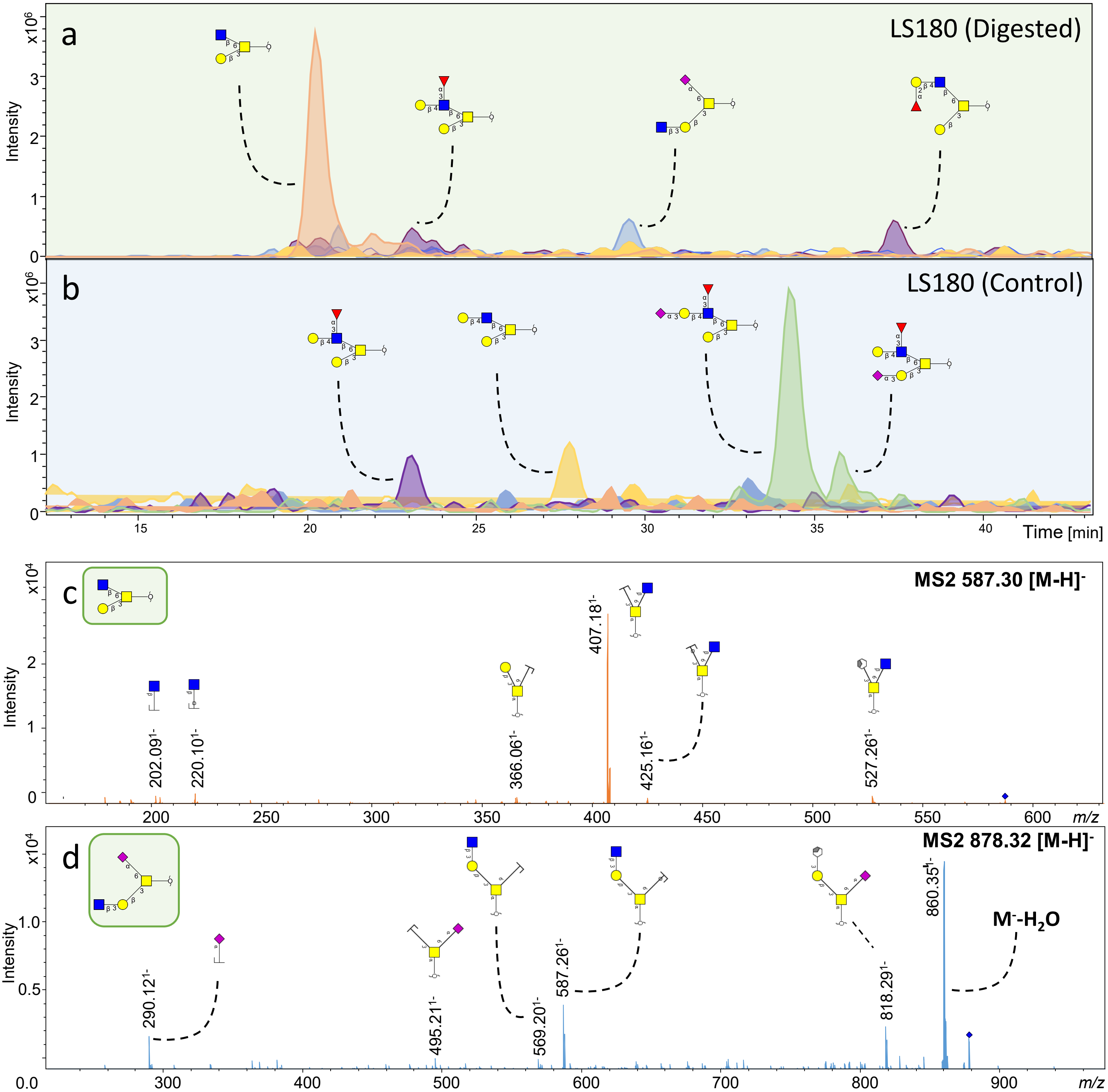

Supplementary figure S6 Glycan linkage determination of Lewis epitopes expressed by LS180 cell line. (**a**) Additional enzymatic cleavage of the α2,3 neuraminidase digested sample was performed with combined α1,3/4 fucosidase and β1,4 galactosidase digestion in order to distinguish between (sialyl) Lewis x and a epitopes. (**a**) A significant increase in intensity of the remaining structures Galβ1-3(GlcNAcβ1-6)GalNAcol at *m/z* 587.30 and GlcNAcβ1-3Galβ1-3(NeuAcα2-6)GalNAcol at *m/z* 878.32 compared to control (**b**) suggest the presence of terminal sialyl-Lewis x epitope Galβ1-4(Fucα1-3)GlcNAc-R on the isomer eluting at RT=34 min. (**d**) The fragmentation of GlcNAcβ1-3Galβ1-3(NeuAcα2-6)GalNAcol revealed the diagnostic ion at *m/z* 495.21 for the α2,6-linked sialic acid to the reducing end GalNAc structures.

CaCo-2 (Control)

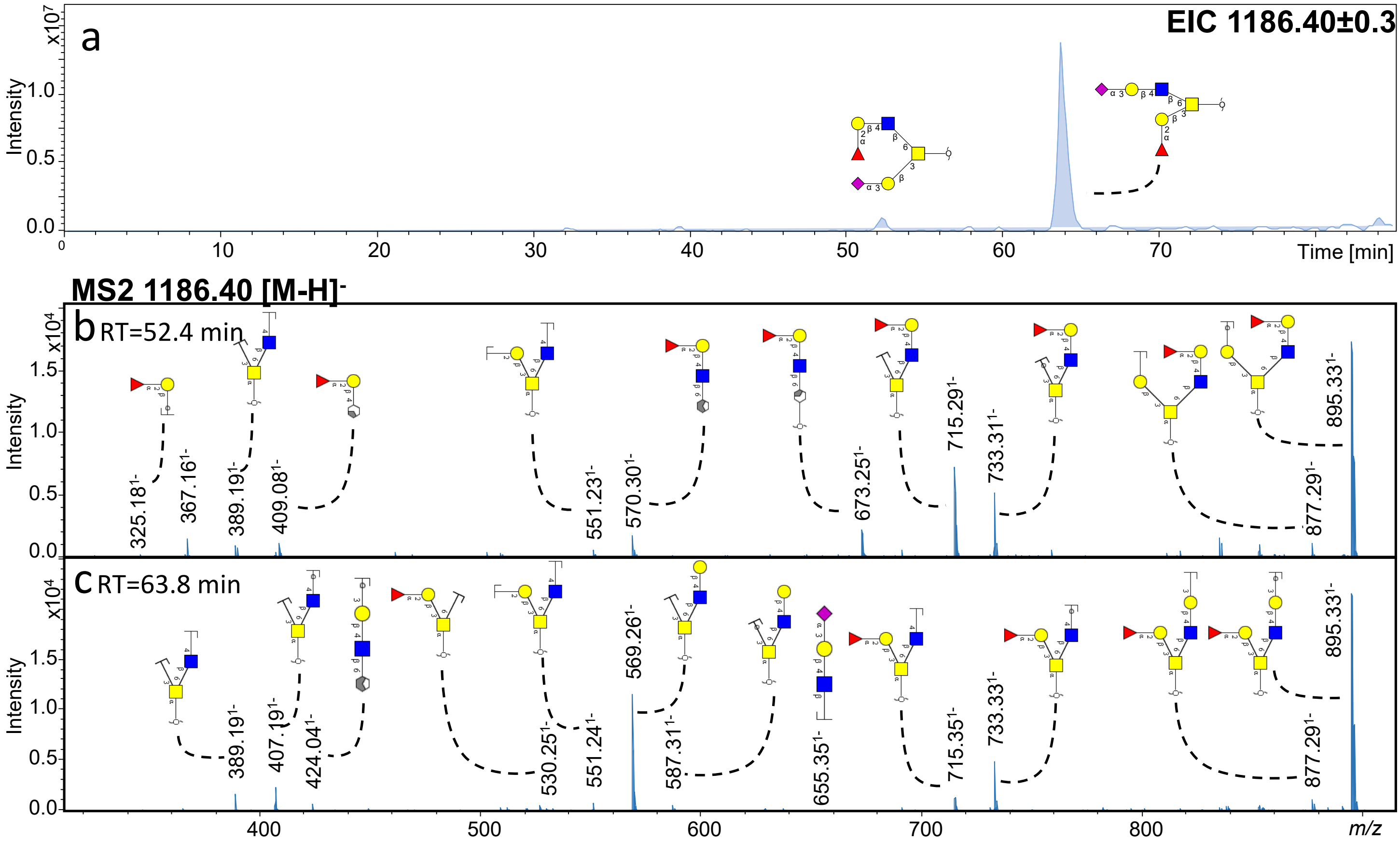

Supplementary figure S7 Glycan linkage determination of major fucosylated glycan structures from CaCo-2 cell line. (**a**) The structures of two isomers present at *m/z* 1186.40 in the sample could be determined from the fragmentation spectra of the sialylated species, by the presence of the diagnostic fragment ion at ^{0,2}A3α-H_2O at *m/z* 409.08 (**b**) confirming the blood group antigen type 2 in the isomer eluting at 52.4 min. (**c**) The ^{0,4}A0α fragment at *m/z* 424.04 of the reducing end GalNAc revealed the composition of the 6 arm of the core 2 isomer at RT 63.8 min, together with the Y1α ion at *m/z* 530.25 indicating the blood group antigen type 3 on the 3 arm.

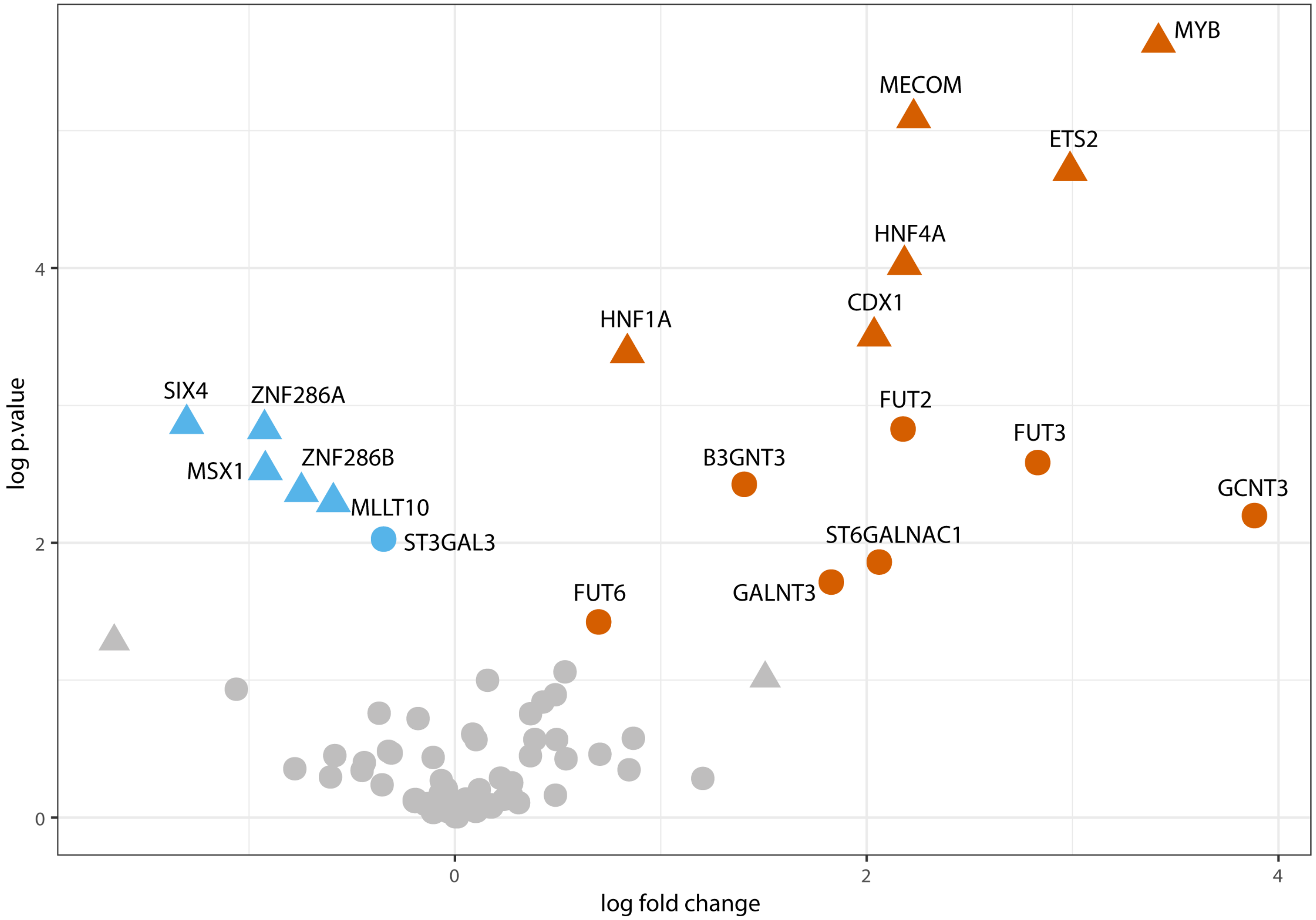

Supplementary figure S8 Differentially expressed glycosyltransferase and transcription factor genes in colon like versus undifferentiated cell lines. Volcano plot displays a selection of genes involved in *O*-glycan biosynthesis (depicted by circles) and transcription factors (depicted by triangles) which show the highest fold change in expression ($\log_e > 0.5$) when comparing colon-like and undifferentiated cell lines with high statistical significance (Bonferroni corrected p-value < 0.05). Genes upregulated in colon like cell lines are marked in red, while genes upregulated in undifferentiated cell lines are marked in blue. [8] The genes that do not pass the thresholds are marked in grey.

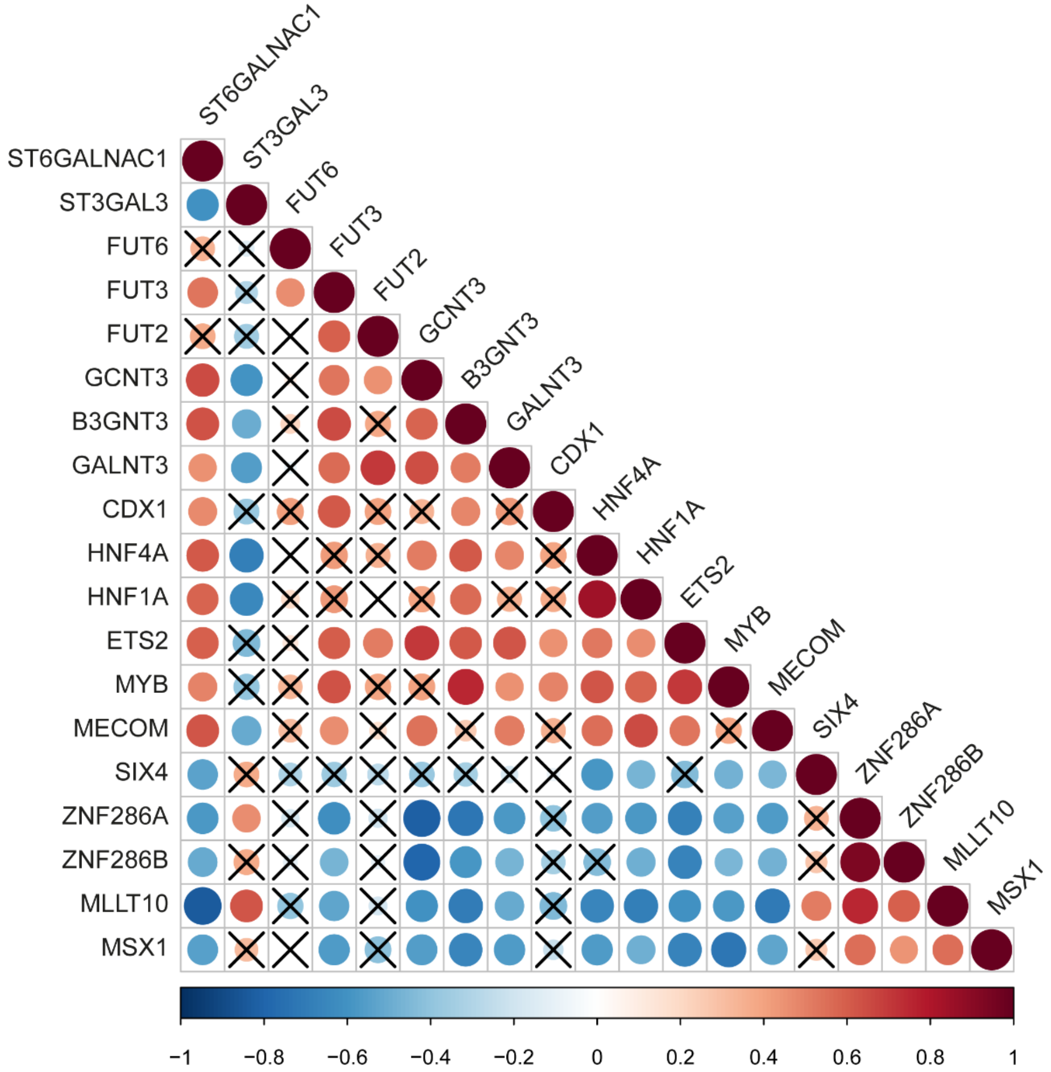

Supplementary figure S9 Correlation between the expression of glycosyltransferase encoding genes and transcription factors. A Spearman's nonparametric correlation was performed with significance α=0.05. Crosses indicate non statistically significant correlations. [8]
